# Cable news and COVID-19 vaccine uptake

**DOI:** 10.1038/s41598-022-20350-0

**Published:** 2022-10-07

**Authors:** Matteo Pinna, Léo Picard, Christoph Goessmann

**Affiliations:** 1 Center for Law and Economics, ETH Zürich, Zurich, Switzerland; 2 Faculty of Business and Economics, University of Basel, Basel, Switzerland

**Keywords:** Psychology and behaviour, Human behaviour

## Abstract

COVID-19 vaccines have reduced infections and hospitalizations across the globe, yet resistance to vaccination remains strong. This paper investigates the role of cable television news in vaccine hesitancy and associated local vaccination rates in the United States. We find that, in the earlier stages of the vaccine roll-out (starting May 2021), higher local viewership of Fox News Channel has been associated with lower local vaccination rates. We can verify that this association is causal using exogenous geographical variation in the channel lineup. The effect is driven by younger individuals (under 65 years of age), for whom COVID-19 has a low mortality risk. Consistent with changes in beliefs about the effectiveness of the vaccine as a mechanism, we find that Fox News increased reported vaccine hesitancy in local survey responses. We can rule out that the effect is due to differences in partisanship, to local health policies, or to local COVID-19 infections or death rates. The other two major television networks, CNN and MSNBC, have no effect. That, in turn, indicates that more differentiated characteristics, like the networks’ messaging or tendency for controversy, matter and that the effect of Fox News on COVID-19 vaccine uptake is not due to the general consumption of cable news. We also show that there is no historical effect of Fox News on flu vaccination rates, suggesting that the effect is COVID-19-specific and not driven by general skepticism toward vaccines.

## Introduction

Since their introduction in late 2020, COVID-19 vaccines have bolstered the fight against the disease, substantially reducing the likelihood of infection and especially severe cases^1–4^. Given their proven effectiveness and the continued social costs of infection, the persistent resistance toward vaccination poses an urgent policy problem. Correspondingly, understanding the determinants of decisions to comply with or resist vaccines poses an urgent scientific question.

There is a small and timely literature providing some initial findings on this question. Exposure to online misinformation is associated with a decline in the willingness to take a COVID-19 vaccine^5,6^. Individuals who are opposed to the vaccine are less likely to obtain information about the pandemic from traditional and authoritative sources^7^.

We add to this research by exploring the role of cable news - a major class of U.S. television stations - in vaccine decisions. In the context of the COVID-19 pandemic, previous work has shown that conservative media consumption is associated with less social distancing^8–10^ and worse COVID-19 health outcomes^11^. These papers on COVID-19 are part of a broader literature on the effects of media on individual preferences and behavior^12^, political elections^13–16^, and local fiscal policies^17^. Because cable news providers vary in their skepticism toward COVID-19 vaccination, differential exposure to their programs might influence reported vaccine hesitancy and observed vaccine uptake. For example, Fox News Channel has been doubtful of scientific consensus and majority expert views^18–20^. In particular, Fox News Channel’s prime time show Tucker Carlson, one of the most popular prime time shows on FNC^21,22^, has taken a strong stance against vaccines, representing deaths following a vaccination to be caused by it, contradictory to available evidence^21,22^.

Our empirical approach pairs data on county-level vaccination rates with data on viewership of the main cable news providers: Fox News Channel (FNC), MSNBC, and CNN. We highlight that these are administrative, rather than survey data, so they are not biased by selective reporting.

In the early months of the vaccination campaign, we do not observe a relationship between cable channel viewership and vaccine uptake. However, in the more recent months starting in May 2021, Fox News viewership becomes negatively related to vaccine uptake. The relationships for the other cable news networks, MSNBC and CNN, remain unchanged.

We can show that the relationship between FNC viewership and lower vaccination uptake is causal using a natural experiment. The networks’ channel position in the lineup provides an exogenous instrument for viewership, as widely used in economics and political science^10,13,16,17,23^. Leveraging the exogenous variation in viewership, we estimate a local average treatment effect and find qualitatively coherent results. Exogenously higher FNC viewership due to channel position causes lower vaccine uptake.

The rest of the paper provides a number of supporting results to understand the most relevant mechanisms. Overall, the results support the interpretation that FNC promulgated a uniquely skeptical narrative about vaccines, that is, a narrative that downplayed their utility and promoted uncertainty on their safety, against the available scientific consensus. That narrative caught on and reduced uptake among the marginal vaccine recipient.

First, we look at the effect of cable news viewership on responses to a national survey, which asked respondents about their hesitancy to take the vaccine. In areas with higher FNC viewership, higher hesitancy to vaccinate was reported. Thus we can provide support for a behavioral mechanism, where FNC’s skeptical vaccine narrative affects vaccination rates by changing attitudes and intentions regarding the vaccine.

Second, we consider whether local health care capacity is driving our results. If that was the case, we should see an effect of Fox News on vaccine uptake also in the early as well as later stages of the vaccination campaign. Yet, we find no effect at all in the early stages while only individuals younger than 65 years old are affected in the later stages. Furthermore, we verify that FNC has no effect on local health care capacity metrics (number of ICU beds/hospitals) or stress metrics (number of COVID-19 infections/deaths).

Having ruled out health care capacity as a potential confounder, we further can infer from the above that the effect of Fox News on vaccine uptake is focused on relatively low-risk individuals (< 65 years) who potentially benefit less from vaccines than older individuals.

Third, we look at partisan affiliation or political ideology as vehicles for differences in beliefs and attitudes about vaccines. It could be that Republicans or conservatives are overall more skeptical of the COVID-19 vaccine, and that the effect of FNC works by increasing the number of Republicans or number of conservatives. Our results show that this is unlikely to be the case, as the effect of FNC on vaccine uptake holds even when controlling for partisan affiliation and political ideology.

Finally, we consider whether FNC has affected general attitudes towards vaccines, for example through anti-science rhetoric. To check this, we look at the effects on seasonal flu vaccines. There is no effect, suggesting that there is no generic anti-vaccine effect and that the effect on COVID-19 vaccines is due to a COVID-specific narrative.

Throughout the paper we address some of the caveats to our analysis: The compliers, i.e., casual television surfers that our instrumental variable analysis is based on, may be different from the general population. In addition, our study is limited to the early phase of the U.S. vaccination campaign during which (1) vaccinations began to be available widely and (2) vaccination certificates played no major role (e.g., to dine inside).

The findings in this paper provide timely insights on the COVID-19 vaccine deployment in the United States. The main cable news television providers are affecting vaccination decisions. Future efforts by government agencies and health organizations to encourage vaccine uptake should account for how media narratives may strengthen or weaken those efforts.

**Figure 1:**
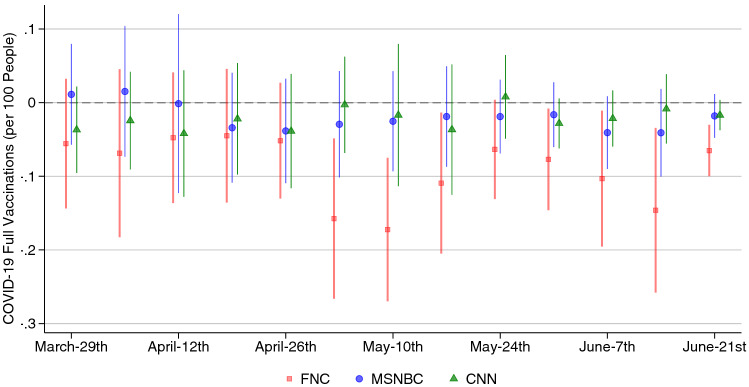
Cable news viewership and weekly vaccination rates, 2021. Coefficient plots with 95% CIs from OLS regressions looking at the association between one standard deviation changes in viewership on weekly vaccinations per 100 people. Regressions include demographic and cable-system controls. Standard errors are clustered by state.

## Main results

In the following, the *final vaccination dose* will refer to the first dose for a single-shot vaccine or the second dose for a two-shot vaccine. Furthermore, we assign weekly data to Mondays for consistency.

Figure 1 shows the association between the viewership of the three major U.S. television networks (FNC, MSNBC, CNN) and the number of people receiving their final COVID-19 vaccination dose within the given week. We obtain those coefficients by running cross-sectional regressions at the county level with 95% confidence intervals. Supplementary Figure S9 additionally provides estimates ranging back to January, based on smaller sample sizes for earlier months, and until the end of 2021.

While we do not observe a statistically significant effect associated with CNN and MSNBC viewership, counties with higher Fox News viewership exhibit a lower percentage of the population getting the vaccine, starting the first week of May.

These estimates may have endogeneity issues, as pre-existing ideologies within counties are likely to be correlated with viewership. Exploiting the fact that casual viewers surfing through channels tend to spend more time watching programs with lower channel numbers, defined as the compliers for the estimated local average treatment effect (LATE), and that these positions in the cable system lineup are exogenously determined, we get around the endogeneity problem by using an instrumental variables approach. We estimate Two-Stage Least-Squares (2SLS) regressions, instrumenting the endogenous viewership with the networks’ channel position. For more details we refer to the “Materials and methods” section.

**Figure 2:**
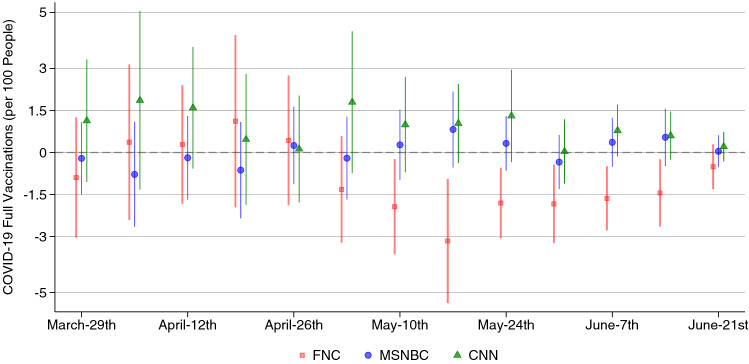
Effect of network viewership on weekly vaccination rates, 2021 (2SLS). Coefficient plots with 95% CIs from 2SLS regressions of the effect of one standard deviation changes in viewership on weekly vaccinations per 100 people. Viewerships are instrumented using the lineup channel positions. Regressions include demographic and cable-system controls. Standard errors are clustered by state. First-stage F-statistics: 13.85 (FNC), 4.45 (MSNBC) and 2.53 (CNN).

Results from the 2SLS analysis (Fig. 2) are qualitatively coherent with the OLS estimates. Starting May 2021, counties with higher Fox News viewership report lower vaccination rates, with a one standard deviation increase in viewership being associated to roughly 1.5–3.2 less vaccinations per 100 people. The effect seems to fade out at the end of June (cf. extended graph in Supplementary Fig. S9). For MSNBC and CNN, with the caveat of a weak first stage, we find a mostly positive effect, though not statistically significant.

In the context of this analysis, a one standard deviation is equal to 2.34 rating points, roughly corresponding to 252 min of weekly viewership for the average household.

Put in perspective, these results imply that watching one additional hour of Fox News per week for the average household (a 30% increase above the mean viewership) reduces the number of vaccinations by 0.35–0.76 per 100 people for the period in which we observe a significant effect.

In the estimation for each network’s effect, our preferred specification accounts for the other networks’ relative channel position and ratings as well as for geographical confounders by using state fixed effects. We weight by the 2010 Census populations, cluster standard errors at the state level and control for counties’ socio-demographic characteristics and political preferences.

These results are robust to a wide range of checks: A smaller and greater set of demographic and U.S. presidential election controls; variations in the instrumentation of the viewership; specification and sample checks controlling for COVID-19 related characteristics of the county as cases and deaths, the ability to handle an outbreak, and surveyed COVID-19 vaccine hesitancy. We refer to the “Materials and methods” section and the Supplementary Materials for further details.

## Analysis of mechanisms

**Figure 3:**
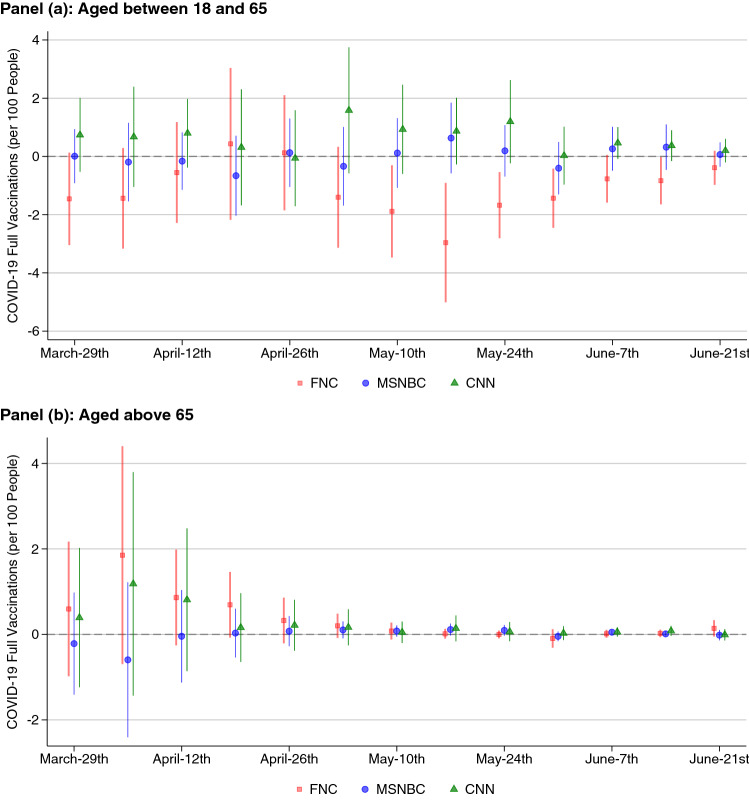
Effect of network viewership on weekly vaccination rates by age group, 2021 (2SLS). Coefficient plots with 95% CIs from 2SLS regressions showing the effect of one standard deviation changes in viewership on weekly vaccinations per 100 people, by age group. Viewerships are instrumented using the lineup channel positions. Regressions include demographic and cable-system controls. Standard errors are clustered by state.

Looking separately at the effect of the networks on vaccinations by age, we observe that results are driven by the share of the population aged 18–65 years, with no significant effect on the group older than 65 years (cf. Fig. 3). It is important to note that, even though the population above age 65 was allowed to get vaccinated starting end of January, roughly 20% of that group took the vaccine in the period of analysis, with still 40% not yet vaccinated.

**Table 1:** Further outcome measures.

	Vacc.	Outbreak	ICU	Hospitals	2016 Flu	2017 Flu	2018 Flu
	Hesitancy	Concern	Beds (#)	(#)	Vacc. (%)	Vacc. (%)	Vacc. (%)
FNC (s.e.)	0.04$^{+}$ (0.02)	0.01 (0.10)	490 (1135)	26 (41)	− 0.58 (7.46)	− 0.45 (7.98)	− 0.05 (8.47)

Two Stages Least Squares (2SLS) estimates of the effect of FNC’s viewership on further outcome measures. Viewerships are instrumented using the lineup channel positions. Regressions include demographic and cable-system controls. Standard errors are clustered by state. Estimates are relative to a one s.d. increase in the network’s viewership. $$^{+}$$ $$p < 0.10$$, $$^{*}$$ $$p < 0.05$$, and $$^{**}$$ $$p < 0.01$$.

We explore four possible mechanisms behind the behavioral effects that we observe.

First, to further investigate the timing of the Fox News effect, we use county-level survey data on hesitancy^24^ about COVID-19 vaccinations for the first half of March 2021. We find that, in areas with higher Fox News viewership, subjects report a higher hesitancy to take the vaccine (Table 1). Together, these results are consistent with the hypothesis that media coverage has reduced vaccinations by discouraging the share of the population with low health-related risks from getting vaccinated.

Second, we look at the the health care system and the COVID-19 situation of the counties. It could be that the areas that have, for example, higher Fox News viewership, systematically differ in terms of the health care system’s capability to handle a COVID-19 outbreak, for example due to effects on local government funding^17^. Or that these counties suffered more severe numbers of cases and deaths in 2020 or the period before the vaccination dates.

To investigate this possible mechanism we run 2SLS regressions on the estimated COVID-19-specific risk of the counties^25^ and the availability of intensive care units (ICUs)^26^ as well as hospitals^26^. We observe no significant effects for any of the networks. As an addition, we replicate our main specification analysis including those outcomes as controls (Supplementary Fig. S17).

Furthermore, our main results are robust to additional controls accounting for the number of COVID-19 cases and deaths one month before the vaccination date or to the overall cumulative cases and deaths or their interaction with the instruments (Supplementary Figs. S15–S16).

Third, Fox News viewership has been shown to be correlated with voting Republican^13,14,16^ and other networks’ viewership might be associated with more anti-vaccination left-wing politics. Following^8^, we show that partisanship is unlikely to be the driver of our results. Our estimates are unaffected when we control for the pre-treatment Republican vote share in 1992 and 1996 (Supplementary Fig. S10) or their interaction with the instruments (Supplementary Fig. S11). In line with that, we are including controls for the pre-pandemic Republican vote share in the 2012 and 2016 elections in our main specification. Our results are furthermore robust to adding self-reported political ideology, partisanship (Supplementary Fig. S12), and their interactions (Supplementary Fig. S13) from the Gallup survey as controls.

The fourth mechanism we explore is anti-science rhetoric. Fox News Channel has been linked to anti-science beliefs^18–20^ and this long-run effect of the network’s viewership might have affected the compliers’ predisposition to vaccinations. Using seasonal flu vaccination data for the sample of Medicare fee-for-service enrollees for the years 2017–2019^27^, we find no significant effect of the networks’ viewership on the percentage of people vaccinated (Table 1 and Supplementary Table S6). We are not fully able to disentangle the effect of these beliefs from the COVID-19 vaccine coverage, because of the limited over-time variation in our channel position instrument. However, if the anti-science rhetoric was the strongest driver of vaccine skepticism, we would expect to see an effect of conservative or liberal media on other vaccination rates.

Overall, the evidence is consistent with FNC having a skeptical narrative, specific to COVID-19, which persuaded marginal vaccine recipients not to take the vaccine. Fox News expressed skeptical views on the risks posed by the Coronavirus at early stages of the pandemic, for example by downplaying the disease as a “normal flu”, ridiculing the “flu panic” and claiming it to be used as “political weapon” against President Trump^8^. Its shows have also been raising doubts about the COVID-19 vaccines^21,22^, for example labeling them as dangerous, or calling out lobbying by “Big Pharma” (Supplementary Fig. S7). In the context of a high stakes decision, individuals might be more susceptible to such strong rhetoric and opinionated hosts, especially if official information lack clarity or coherence. Our results, in addition to the previous literature on slanted media and behavioral responses to the pandemic, suggest that the COVID-19 coverage of Fox News is at least partially responsible in reducing participation in vaccination efforts, likely in addition to the effect of the persistent anti-science slant.

## Conclusions

Our results show that Fox News is reducing COVID-19 vaccination uptake in the United States, with no evidence of the other major networks having any effect. We first show that there is an association between areas with higher Fox News viewership and lower vaccinations, then provide an instrumental variable analysis to account for endogeneity, and help pin down the magnitude of the local average treatment effect. Supporting analysis suggests that media emphasis on minority viewpoints against scientific consensus is linked to vaccination hesitancy, producing significant behavioral effects in the share of the population that is younger than 65 years with low health risks.

Overall, an additional weekly hour of Fox News viewership for the average household accounts for a reduction of 0.35–0.76 weekly full vaccinations per 100 people during May and June 2021. This result is not only driven by Fox News’ anti-science messaging, but also by the network’s skeptic coverage of COVID-19 vaccinations.

## Materials and methods

This section provides a more detailed description of the data and methods used in the paper. The study was conducted in accordance with the relevant guidelines and regulations.

### Data sources

Data on vaccinations, the main focus of analysis, are provided by Centers for Disease Control and Prevention (CDC). Data are available for roughly 2750 counties from 47 states, for the main period of analysis. While we use the share over the total population to analyse vaccinations by age, the results remain unchanged when dividing by the available approximate share of the population in that age group (e.g. older than 60).

Pivotal for our empirical strategy are data on the U.S. Broadcast media provided by Nielsen. We use channel lineup positions for FNC, MSNBC and CNN from 2016, the latest year of data availability. Viewership is expressed in ratings, the number of minutes that each household tuned in to each specific channel during the months of January and February 2020. Throughout the analysis we use demographics from the 2010 U.S. Census and data on political attitudes from the U.S. presidential elections.

While investigating the mechanism behind the observed effects and their robustness, we use several other data sources. For political party self-identification and ideology from 2012 to 2020, we use data from the Gallup Polling Social Series. Moreover, we also look at overall television viewership measures using the American Time Use Survey for the period 2010–2019. To account for locality differences in the health care system and the COVID-19 situation, we use data from KHN on the number of ICU units and Hospitals and data from the New York Times for COVID-19 cases and deaths. We use survey measures of hesitancy towards the COVID-19 vaccine and data on the counties’ ability to handle a COVID-19 outbreak from the CDC for March 2021. Flu vaccination data based on Medicare fee-for-service enrollees for the years 2017–2019 come from the County Health Rankings & Roadmaps program of the University of Wisconsin.

### Association between networks’ viewership and COVID-19 vaccinations

In Fig. 1, we look at the county-level association between FNC, MSNBC and CNN viewership and the weekly number of vaccinations per 100 people. Our estimation approach consists of a series of cross-sectional regressions run separately for each week. We use state fixed effects to account for time-invariant characteristics at the state level, weight by county population and include county-level socio-demographic characteristics for population density, land area, working-age, eligibility for food stamps, sex, Black, white, and below high-school-educated or college-educated. Standard errors are clustered at the state level. For each network, we control for the other two networks’ viewership and their relative channel position (defined as the difference between each other network and the network of interest). Results are robust to exclusion of these controls.

### Causal effect of networks’ viewership on COVID-19 vaccinations

In order to investigate the causal effect of viewership, we employ an instrumental variable approach first proposed by^16^, using the network’s channel position in the cable system lineup as an instrument for viewership. Our analysis follows closely the one from^8^, who study the effect of Fox News on mobility during COVID-19. We expand the focus of the research by looking into two other major networks in detail: CNN and MSNBC. To better assess the relative effect of each network, we make use in the analysis of the relative channel position between the networks in a similar fashion as^17^.

First, we take the channel position of the network with the highest viewership (FNC) as a reference point, and calculate the relative position by subtracting it from the channel position of CNN and MSNBC. We translate this into a Two-Stage Least-Squares estimation, by predicting the viewership of the reference network FNC with its channel position, controlling for its relative position to the other networks, and instrumenting the viewership of CNN and MSNBC with the position relative to both other networks.

Using the relative channel position in the instrumentation allows us to partially improve the weak first stage prediction of viewership for CNN and MSNBC, a problem encountered in previous works^13,16^, that also represents a caveat to our analysis. For completeness, we report our main results using the standard estimation strategy from^16^ in Supplementary Fig. S6. Results are virtually identical for FNC and CNN, while the MSNBC model becomes noisy.

The 2SLS estimation follows the same specification as the OLS estimation and is furthermore used to investigate the additional outcomes reported in Table 1. We check then for instrumental relevance, i.e. that the channel position predicts viewership. Supplementary Figure S3 shows the negative correlation between channel positions and viewership, while Supplementary Table S3 shows the first stage for our instrumentation employing the relative channel position of the networks. We run, finally, a series of tests to verify if our instruments are exogenous to a range of predetermined county characteristics that could be correlated with vaccinations. These regressions (see Supplementary Table S4), suggest that the instrument is indeed exogenous to such characteristics.

### Robustness checks

We present a wide set of robustness checks to support our analysis.

First, we show that our specification is robust to various changes in the set of controls and that estimates are not driven by specific outlier states (Supplementary Fig. S23).

Second, we run checks on the unbalanced characteristics and see that our results are robust to adding polynomials of these variables or interacting them with the instruments (Supplementary Figs. S4–S5).

Third, we replicate our main results by adding as controls, or interacting with the instrument, the relative share of Republican votes in the U.S. presidential elections from 1992 and 1996 (Supplementary Figs. S10–S11). To further support the fact that our results are not driven by partisanship, we use data on self-reported party affiliation and ideology from the Gallup Polling Social Series and show that our results are robust to their inclusion as controls or as interactions with the instrument.

We additionally replicate^8^ using data on TV viewership from the American Time Use Survey. From that analysis, we find that networks don’t have an effect on total hours of TV watched (Supplementary Fig. S14) and that our estimates do not vary with its inclusion as control.

Finally, we show that the effect on vaccinations is not driven by the recent number of COVID-19 cases or the overall number of cumulative cases and deaths (Supplementary Figs. S15–S16).

## Supplementary Information

Supplementary Information.

## Data Availability

The replication data for this paper is available from the ETH Zurich Research Collection via 10.3929/ethz-b-000569576. Due to licensing restrictions we had to remove variables stemming from Nielsen Media Research, the Gallup Polling Social Series, and Kaiser Health News from the replication package. These data can be obtained separately from the respective institutions. The replication package and the supplementary material include detailed documentation on the data sources.
