# Supplementary material for: Cable news and COVID-19 vaccine uptake
Source: Sci Rep. 2022 Oct 7;12:16804. doi: 10.1038/s41598-022-20350-0 (PMC9540283; doi:10.1038/s41598-022-20350-0)
Supplement: Supplementary file 1 — Supplementary Information. [file 41598_2022_20350_MOESM1_ESM.pdf]

Supplementary Materials

Contents

S1 Further information on data sources **2**
S1.1 **Channel positions** . . . 2
S1.2 **Channel viewership** . . . 2
S1.3 **COVID-19 cases and fatalities** . . . 2
S1.4 **COVID-19 vaccinations** . . . 2
S1.5 **COVID-19 vaccination hesitancy** . . . 2
S1.6 **Heath care data** . . . 3
S1.7 **Data on the counties' ability to adequately react to COVID-19** . . . 3
S1.8 **Seasonal flu vaccination** . . . 3
S1.9 **Data on the counties' ability to adequately react to COVID-19** . . . 3
S1.10 **Demographics and politics** . . . 3
S1.11 **Further Data** . . . 3

S2 Summary statistics **4**
S2.1 **Weekly evolution of COVID-19 in the U.S.** . . . 4
S2.2 **Summary statistics for all variables** . . . 6

S3 Instrument validity **8**
S3.1 **First stage** . . . 8
S3.2 **Placebo checks** . . . 10
S3.3 **Alternative IV approach** . . . 13

S4 Tables for main results **14**

S5 Other outcomes for all networks **14**

S6 Networks discourse **15**

S7 Robustness checks **16**
S7.1 **OLS estimates for main results** . . . 16
S7.2 **Results with extended period of analysis** . . . 17
S7.3 **Partisanship and ideology checks** . . . 18
S7.4 **Television viewership checks** . . . 22
S7.5 **Health and health care sector checks** . . . 23
S7.5.1 COVID-19 cases and fatalities . . . 23
S7.5.2 Vaccine hestiancy and outbreak risk . . . 25
S7.5.3 Number of ICU beds and hospitals . . . 26
S7.5.4 Influenza vaccinations . . . 27
S7.6 **Specification checks** . . . 28
S7.7 **Sample checks** . . . 31

S1 Further information on data sources

This appendix section provides additional information about our data. Summary statistics are reported in Table S1.

S1.1 Channel positions

Channel positions for FNC, MSNBC and CNN come from the Nielsen FOCUS database, which reports channel lineups of all U.S. local broadcast systems, with information about the area served by the system at the zipcode level. We use channel positions from 2016, the latest year for which we have access. We aggregate the data at the county level, by averaging zipcode-level channel positions with weighting by population size. To address the presence of outlier channels, we winsorize the variables at the top and bottom deciles.

S1.2 Channel viewership

Television viewership by county of FNC, MSNBC and CNN, is provided by Nielsen. The measure is"ratings," which is proportional to the number of minutes that each household tuned in to each specific channel during the months of January and February 2020. We standardize the viewership throughout the paper by its standard deviation for all networks. Some counties in the raw data were split in parts (e.g. North County-A, East County-A), and were aggregated together by simple average.

S1.3 COVID-19 cases and fatalities

Confirmed COVID-19 cases and fatalities are from the The New York Times (https://github.com/nytimes/covid-19-data). The dataset is already at the daily and county level and starts in January 2020. As these variables are expressed in a cumulative manner, therefore we generate new daily cases and fatalities by subtracting observations of day $n-1$ from day n.

We further calculate weekly estimates by summing over daily estimates by calendar weeks. The New York Times states that cumulative cases can sometimes decrease after a state corrects a mistake in reporting. When we observe such a correction, we set weekly observations to missing if the weekly sum of daily observations is negative, too.

S1.4 COVID-19 vaccinations

The COVID-19 vaccine roll out in the United States began in December 2020, following federal guidelines and CDC recommendations (state governments had the possibility to adjust the vaccination strategy depending on their demographic, health care system, and COVID-19 situation): First, healthcare workers were vaccinated (starting December 2020), then people aged over 65 (end of January 2021), people with medical conditions and disabilities (mid of March 2021), and people aged above 50 (April 2021). This was finally followed by an expansion of eligibility to virtually the whole population by May 2021 (https://www.cdc.gov/coronavirus/2019-ncov/vaccines/recommendations-process.html). Some states even started to distribute the vaccine to all adults in late March (e.g. Alaska, Georgia, Mississippi, Ohio, and Texas).

The statistics on full COVID-19 vaccinations (final doses) come from the Centers for Disease Control and Prevention (CDC), who aggregate data reported by state health agencies, jurisdictions and federal entities. The CDC data contain county-level numbers for most U.S. states and territories. Statistics for Texas and Hawaii are only reported at the state level – we thus do not include those states in our analysis. Also, California does not report data on counties with fewer than 20,000 inhabitants, so we cannot include those counties in our analysis. Taken together, the vaccination data include roughly 2950 counties, of which around 2750 are used for the main analysis. This difference is due to a lack of data on viewership, for example for the states/territories of Alaska, Guam, Puerto Rico, and the Virgin Islands.

Data access is possible via CDC's dedicated API. We collect information for the first of January 2021 and onwards. Similarly to the COVID-19 case and fatality numbers, vaccination data is provided in a cumulative manner. Generating Monday-to-Sunday weekly vaccinations, we end up with weekly data starting January 11. The latest data (until end of 2021) have been downloaded with the same methodology in April 2022.

Vaccination estimates refer to full vaccinations, which generally comprise two doses spaced by 4 to 5 weeks unless only one dose is required (e.g. J&J/Janssen vaccine). The CDC neither account for the timing between the two doses, nor for the delay after which the vaccine becomes effective. Note that counties refer to the individual's county of residence, not the county where they got vaccinated. Data is available for the total population, for adults aged between 18 and 64 years old, and for adults aged 65 or older.

S1.5 COVID-19 vaccination hesitancy

Vaccination hesitancy data originate from the Census Bureau's Household Pulse Survey (HPS) conducted in the first half of March 2021. In the survey, respondents were asked to answer to "Once a vaccine to prevent COVID-19 is available to you, would you ... get a vaccine?" with: (1) "definitely get a vaccine", (2) "probably get a vaccine", (3) "probably not get a vaccine", (4) "definitely not get a vaccine."

The CDC use the HPS to estimate hesitancy rates at the state level, followed by an estimation at the Public Use Microdata Areas (PUMA) level using the Census Bureau's 2019 American Community Suvery 1-year Public Use Microdata Sample. Then, county estimates are generated using the Missouri Census Data Center PUMA-to-county crosswalk. For PUMAs overlapping with multiple counties, data is averaged using the 2010 Census populations. This data, we retrieved from the CDC's website https://data.cdc.gov.

S1.6 Heath care data

Data on ICU units and the number of hospitals for 2438 counties are provided by Kaiser Health News[1] (KHN). Bed counts stem from the hospitals' financial cost reports, filed annually to the Centers for Medicare & Medicaid Services. For more information on their methodology and data sources check: https://khn.org/MTA2ODgzMw.

S1.7 Data on the counties' ability to adequately react to COVID-19

The CDC report a variable measuring the counties' ability to handle a COVID-19 outbreak, ranging from 0 to 1 with 1 being the most vulnerable. Data originates from the Surgo COVID-19 Vaccine Coverage Index (CVAC), which is based on measures for access to health care, affordable housing, transportation, childcare, and safe and secure employment to predict how well counties can handle a COVID-19 outbreak.

S1.8 Seasonal flu vaccination

To our knowledge, there is no county level dataset on seasonal flu vaccination that covers all individuals in the United States. We use flu vaccination rates among Medicare fee-for-service enrollees, provided by the County Health Rankings & Roadmaps program from the University of Wisconsin. They take data from the CMS Office of Minority Health's Mapping Medicare Disparities (MMD) data tool that reports various health outcomes at the county level. The influenza vaccination prevalence rates are calculated by searching for the respective diagnosis code in Medicare beneficiaries' claims and dividing the sum by all Medicare beneficiaries in counties. Other recent papers also use this data source in the COVID-19 context (e.g.[2]).

We take county level data for all 50 states from the 2019, 2020 and 2021 reports, each containing estimates for the year three years before the report date. Therefore, in our analysis we can integrate the flu vaccination rates of fee-for-service enrollees for the years 2016, 2017 and 2018. We understand that this data is a sub-sample of the U.S. population that have access to Medicare. In 2019, 58 million Americans were covered by Medicare, which represents health coverage benefits for most adults aged 65 and older (Census Bureau, Health Insurance Coverage in the United States: 2019, issued September 2020).

S1.9 Data on the counties' ability to adequately react to COVID-19

The CDC report a variable measuring the counties' ability to handle a COVID-19 outbreak, ranging from 0 to 1 with 1 being the most vulnerable. Data originates from the Surgo COVID-19 Vaccine Coverage Index (CVAC), which is based on measures for access to health care, affordable housing, transportation, childcare, and safe and secure employment to predict how well counties can handle a COVID-19 outbreak.

S1.10 Demographics and politics

Other local socio-economic variables come from the 2010 U.S. Census. These variables are all at the county level and include, following the specification in[3]: *population*, *population density*, *land area*, *working-age share* of population aged 20-69 over other ages, proportion eligible for *food stamps*, proportion who never attended *no high school*, proportion who attended *college*, a dummy for an above-median *Black population share*, a dummy for an above-median *white population share*, and the proportion of *males*.

We also use data on the *Republican vote share* of the 2012 and 2016 presidential elections. We further expand this set of controls in the robustness check by furthermore including from the 2010 U.S. Census: proportion who attended *high school*, a dummy for above-median *Hispanic population share*, a dummy for above-median *Asian population share*, proportion belonging to *middle income categories* (20-25k, 25-30k, 30-35k, 35-40k, 40-45k, 45-50k), proportion working in *occupation categories* (management and professional, services, sales and office, construction, extraction and maintenance, production, transportation and material moving). In the supplementary materials robustness checks, we complement the Census variables with further occupation shares from the 2019 American Community Survey (ACS), which are also provided by the Census Bureau. The ACS occupation shares comprise the medical, retail, agriculture, industrial, and transport sectors.

S1.11 Further Data

Other data sources are used in the supporting analysis. **Gallup Polling Social Series**: political party self-identification and ideology for the years 2016-2020. Television viewership data from the **American Time Use Survey** for the period 2015-2019 (338 counties).

S2 Summary statistics

S2.1 Weekly evolution of COVID-19 in the U.S.

In Figure S1, we report weekly full vaccinations per age group in our sample of counties, and the average percentage of fully vaccinated individuals by age group. Vaccination efforts began in mid-December 2020 for the elderly and healthcare workers. We observe a sharp increase in full vaccinations for the week of April 12th, one month after several states began to open vaccination to all adults. Figure S2 reports the number of COVID-19 confirmed cases and fatalities for the same set of counties.

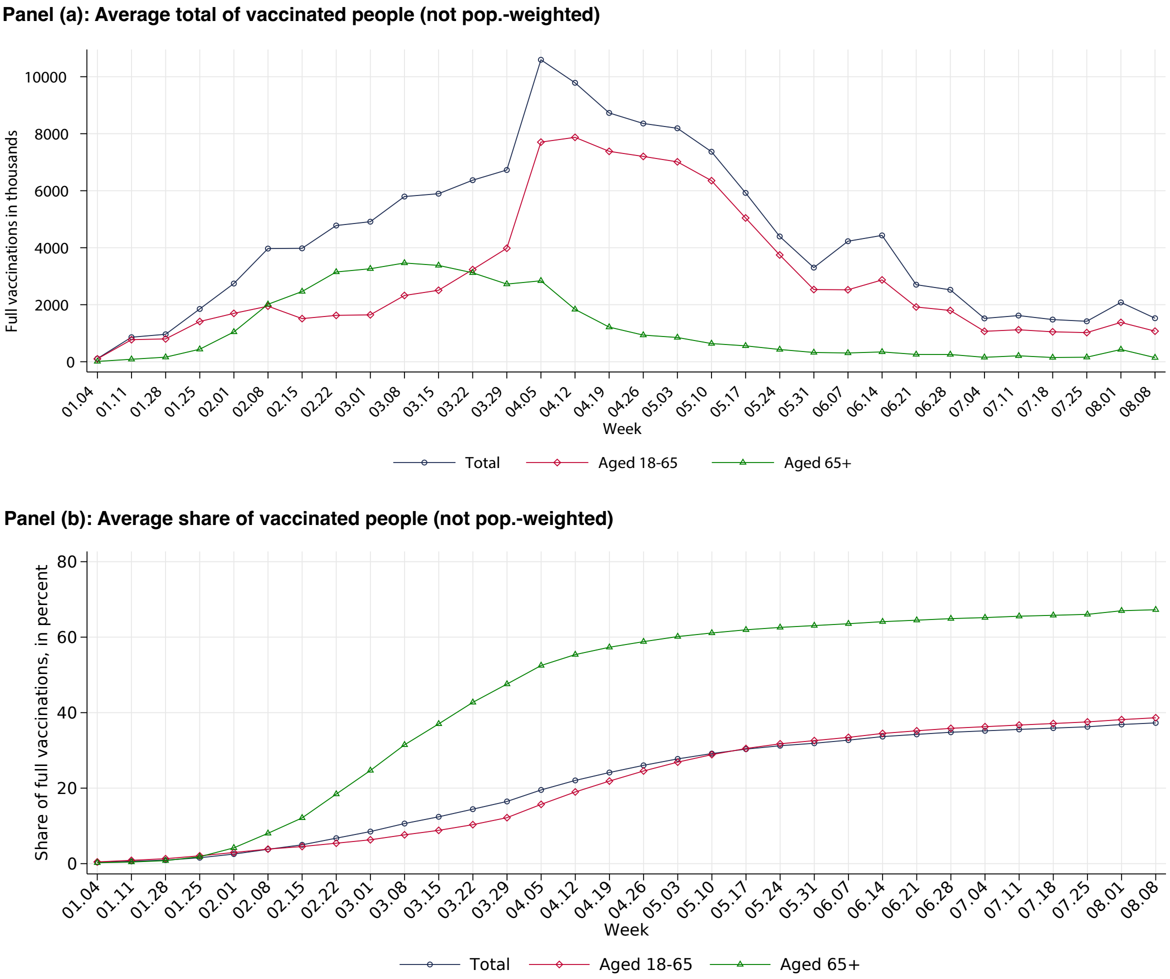

Figure S1. Full vaccinations in our analysis sample of counties, 2021.

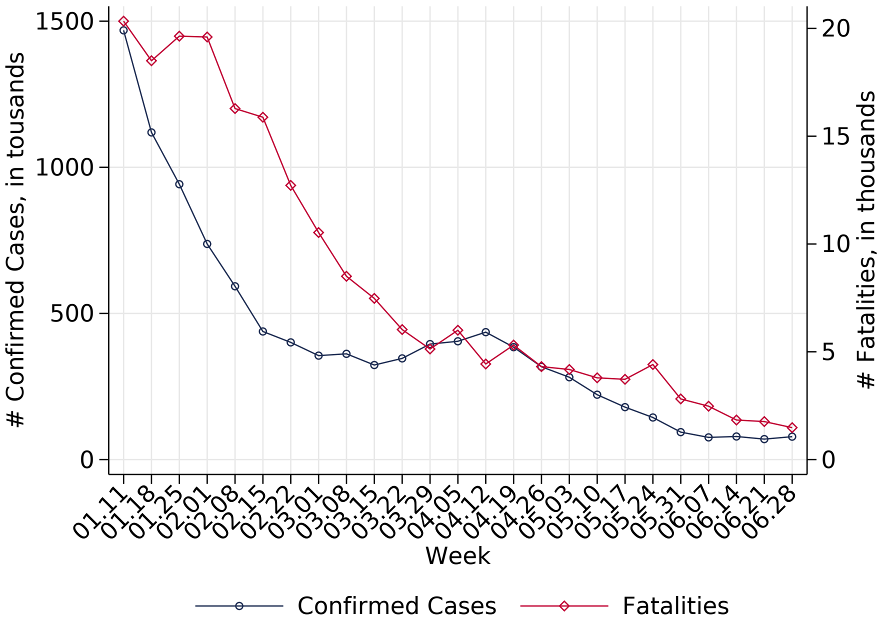

Figure S2. Average number of confirmed COVID-19 cases and fatalities, 2021 (not pop.-weighted).

S2.2 Summary statistics for all variables

Variable	Mean	Std. Dev.	Min.	Max.	N
News channels					
FNC % of ratings (2020)	1.655	2.342	0	67.400	3042
MSNBC % of ratings (2020)	0.537	0.834	0	26.4	3042
CNN % of ratings (2020)	0.383	0.631	0	20.3	3042
FNC channel position (2016)	74.292	38.373	31.43	140.109	3036
MSNBC channel position (2016)	82.901	42.874	34.946	158.798	2999
CNN channel position (2016)	65.13	37.346	24.05	129.149	3039
% pop. zipcodes w/ access to FNC (2016)	0.92	0.164	0	1	3081
% pop. zipcodes w/ access to MSNBC (2016)	0.896	0.208	0	1	3044
% pop. zipcodes w/ access to CNN (2016)	0.925	0.159	0	1	3084
ATUS data (minutes average - 2019)	116.829	23.154	45	260	338
Demographic					
Population	100526.181	317471.581	80	9818535	3042
Land area	511.685	664.989	1.553	9309.787	3042
Population density	187.898	919.263	0.129	33886.035	3042
Age imbalance	1.703	0.226	1.09	4	3042
% Aged 18+	0.765	0.032	0.607	0.902	3042
% Eligible food stamps	0.126	0.06	0	0.431	3042
% White	0.833	0.161	0.121	0.991	3042
% Black	0.089	0.144	0	0.851	3042
% Asian	0.011	0.023	0	0.439	3042
% Hispanic	0.081	0.127	0.003	0.958	3042
% Male gender	0.499	0.021	0.438	0.719	3042
% Without High School education	0.163	0.071	0.014	0.537	3042
% High School graduate	0.354	0.069	0.091	0.786	3042
% College graduate	0.192	0.085	0.054	0.706	3042
% Income 20 to 25 thousands	0.064	0.017	0	0.165	3042
% Income 25 to 30 thousands	0.061	0.015	0.014	0.157	3042
% Income 30 to 35 thousands	0.059	0.014	0	0.13	3042
% Income 35 to 40 thousands	0.054	0.013	0	0.134	3042
% Income 40 to 45 thousands	0.053	0.012	0	0.138	3042
% Income 45 to 50 thousands	0.046	0.011	0	0.105	3042
Political					
% Republican (1992 Pres. Elections)	0.504	0.112	0.135	0.892	3104
% Republican (1996 Pres. Elections)	0.505	0.117	0.107	0.882	3104
% Republican (2012 Pres. Elections)	0.597	0.146	0.072	0.959	3039
% Republican (2016 Pres. Elections)	0.639	0.153	0.083	0.953	3042
% Republican affiliation (Gallup 2012-2019)	0.586	0.253	0	1	2972
% Conservative affiliation (Gallup 2012-2019)	0.487	0.253	0	1	2983
% Republican affiliation (Gallup 2016-2019)	0.597	0.306	0	1	2703
% Conservative affiliation (Gallup 2016-2019)	0.476	0.307	0	1	2725

Table S1. Summary statistics (1/2).

Variable	Mean	Std. Dev.	Min.	Max.	N
Occupation					
% Management and Professional	0.302	0.061	0.113	0.672	3042
% Services	0.179	0.034	0.074	0.387	3042
% Sales and Office	0.227	0.032	0.07	0.368	3042
% Construction	0.046	0.023	0.008	0.22	3042
% Production	0.16	0.057	0.013	0.368	3042
% Arts, Design, Entertainment, Sports, and Media	0.038	0.02	0.012	0.264	3042
% Medical (ACS)	0.05	0.01	0.027	0.142	430
% Retail (ACS)	0.059	0.009	0.031	0.091	430
% Agriculture and Farming (ACS)	0.004	0.007	0	0.067	430
% Manual and Industrial (ACS)	0.077	0.023	0.017	0.158	430
% Transport (ACS)	0.021	0.006	0.006	0.053	430
Health care and COVID-19					
# ICU Beds	30.473	94.968	0	2126	2438
# Hospitals	1.678	2.872	0	76	2438
CVAC Level of concern about a COVID-19 outbreak	0.5	0.289	0	1	3142
% COVID-19 Vaccine Hesitance (Surveyed)	0.191	0.053	0.05	0.323	3142
% Influenza vaccination (2016)	40.481	9.742	3	65	3126
% Influenza vaccination (2017)	41.741	9.775	4	66	3124
% Influenza vaccination (2018)	43.078	10.01	4	67	3124

Table S2. Summary statistics (2/2).

S3 Instrument validity

We check here the two assumptions that are made for every IV analysis: relevance and exogeneity.

S3.1 First stage

First, we check for relevance, i.e. that the instruments – the networks' channel positions – are correlated with the networks' viewership. Figure S3 shows the baseline negative correlation between channel position instrument and the relative network viewership, meaning that a lower channel position in the lineup is associated with higher viewership.

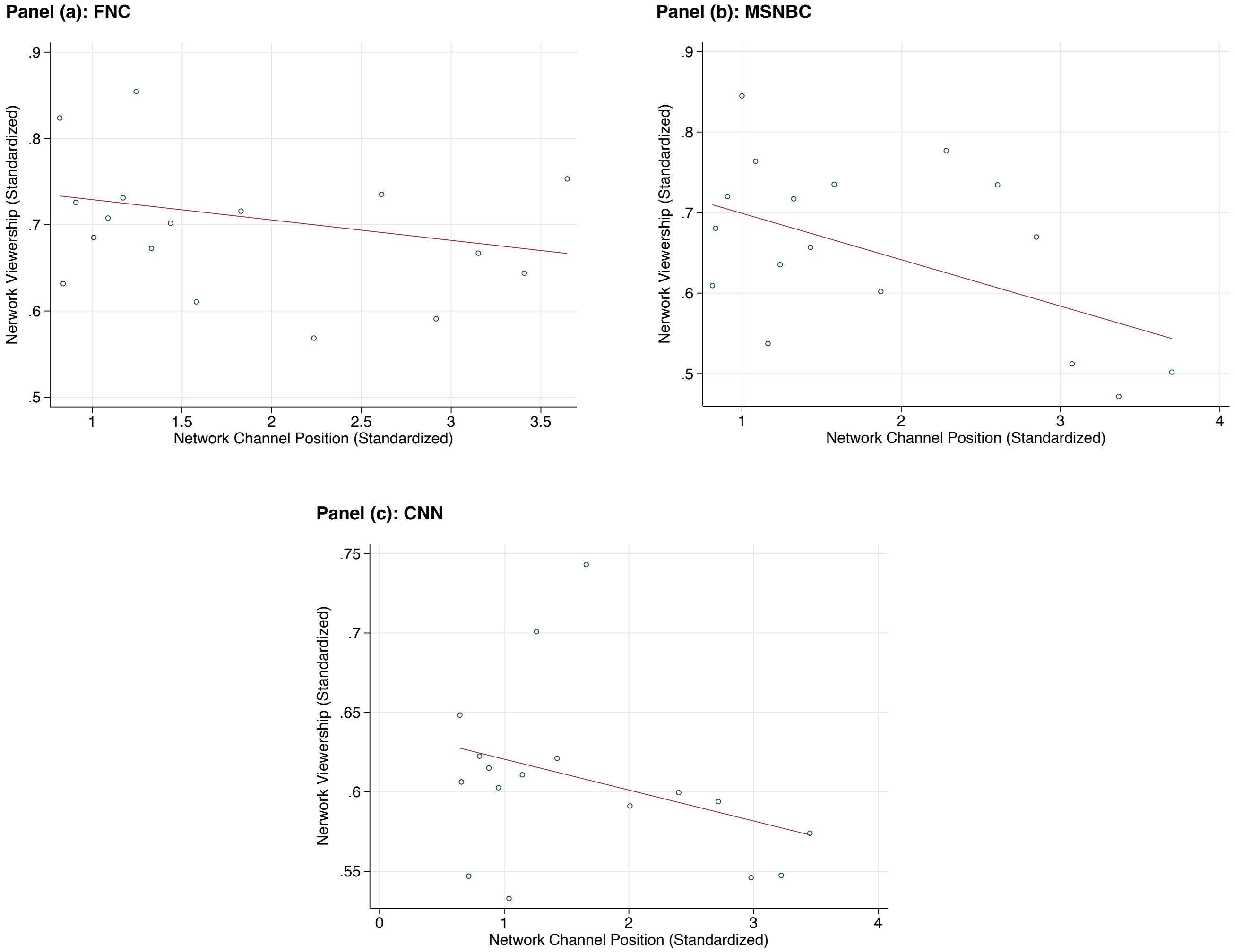

Figure S3. Binned scatterplots for the correlation between viewership and channel position.

We attempt to address the problem of a weak first stage – as faced in previous literature[3,4] for the networks CNN and MNBC – by accounting for the relative lineup position of the networks. For that purpose, we are taking the channel position of the network with highest viewership, FNC, for the calculation of the other networks' relative positions. While this alternative instrumentation improves the prediction of the viewership measures slightly, we still observe a weak first-stage F-statistic of 4.45 for MSNBC and 2.53 for CNN, and warn the readers of this caveat in our analysis in the main text. Coherent with other studies, we obtain a strong first-stage F-statistic of 13.85 for the Fox News Channel. We furthermore show in Figure S6, that our results are virtually identical for FNC and CNN when using the standard instrumentation approach – of directly using the network's channel position on its viewership – but become noisy for MSNBC – with a first-stage F-statistic of 0.2.

Channel Position	FNC viewership	CNN viewership	MSNBC viewership
FNC	-0.04** (0.01)	—	—
CNN-FNC	—	-0.15* (0.07)	—
CNN-MSNBC	—	0.04 (0.03)	—
MSNBC-FNC	—	—	0.12* (0.06)
MSNBC-CNN	—	—	-0.17** (0.06)
Kleibergen-Paap rk Wald F-statistic	13.85	2.52	4.45

Table S3. First stage with the cable network's relative channel position. Variables are standardized by their standard deviation. * $p < 0.05$, and ** $p < 0.01$.

S3.2 Placebo checks

Second, we run checks to support the hypothesis that channel positions are not correlated with local characteristics of the counties that could influence the vaccination decision. A series of empirical checks confirm that the instrument is exogenous (cf. Table S4). For the main specification, we observe that, out of our five different instruments, three instruments each have only one variable showing a correlation with significance at the 5% level: above median White population for MSNBC-FNC, share of the population with education lower than high school for CNN-FNC, and land area for FNC.

Variable	FNC	CNN-FNC	CNN-MSNBC	MSNBC-FNC	MSNBC-CNN
Age imbalance	0.0201	-0.119	-0.0668	0.0368	0.0664
	(0.0284)	(0.128)	(0.0463)	(0.0446)	(0.0459)
Food stamps	0.0756	-0.123	-0.0711	0.0366	0.0679
	(0.0391)	(0.121)	(0.0651)	(0.0455)	(0.0665)
College	-0.0685	0.243	0.0909	-0.0164	-0.0962
	(0.0373)	(0.147)	(0.0596)	(0.0478)	(0.0607)
Male	-0.0261	-0.0117	0.000347	-0.00660	-0.00104
	(0.0198)	(0.0635)	(0.0342)	(0.0289)	(0.0353)
No high school	-0.0294	0.168*	0.0837	-0.0335	-0.0802
	(0.0263)	(0.0743)	(0.0449)	(0.0515)	(0.0465)
Black pop. above median	-0.0631	0.0416	0.0521	-0.0424	-0.0506
	(0.0472)	(0.130)	(0.0710)	(0.0626)	(0.0708)
White pop. above median	0.0496	0.0971	-0.0645	0.0969*	0.0601
	(0.0312)	(0.0974)	(0.0481)	(0.0478)	(0.0486)
Population density	-0.0543	0.12	0.0824	-0.0498	-0.0875
	(0.0286)	(0.109)	(0.0521)	(0.0260)	(0.0574)
Area	0.0432*	0.0507	0.0297	-0.0162	-0.0316
	(0.0173)	(0.0389)	(0.0243)	(0.0195)	(0.0257)

Table S4. Balance checks on channel position instruments. We report in the table above a correlation check between our instruments and local characteristics. We run reduced form regressions with the specified characteristic as the outcome and other controls still on the right-hand side. Coefficients are standardized by the standard deviation. * $p < 0.05$, and ** $p < 0.01$.

We investigate these unbalanced characteristics by checking if they are systematically correlated with the outcome of interest. We include these characteristics as controls in our main specification and we furthermore verify our results to be robust when adding them as polynomials (Figures S4) or when interacting them with the instruments (Figures S5).

Panel (a): Polynomials for % White

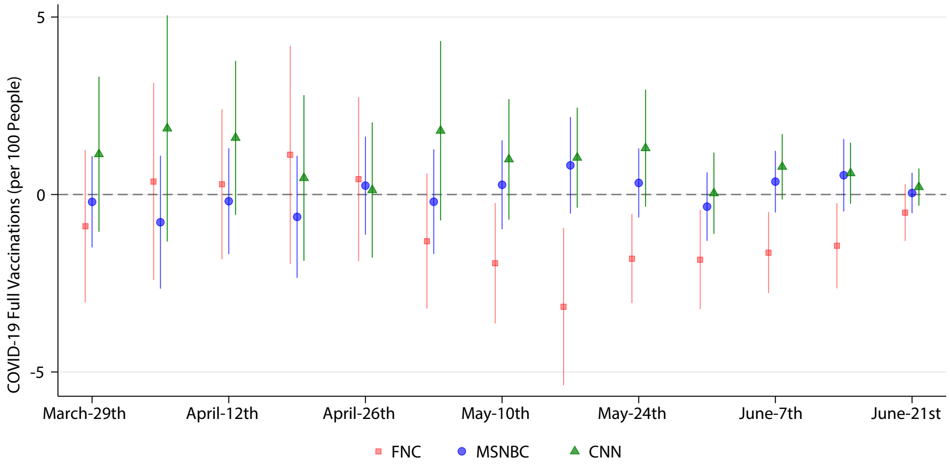

Panel (b): Polynomials for % with no high school education

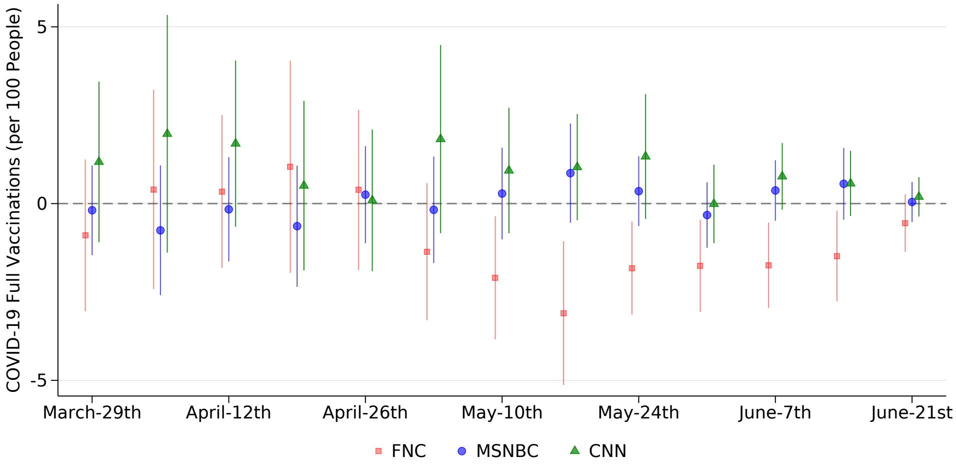

Panel (c): Polynomials for land area

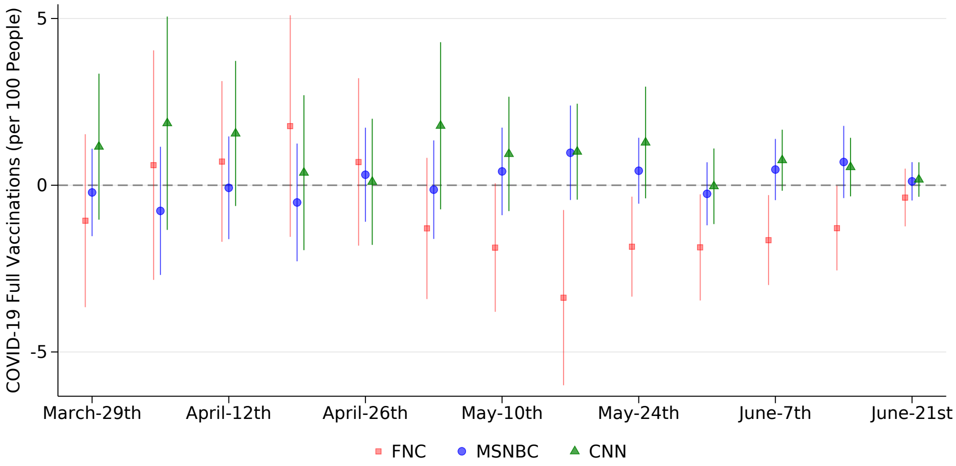

Figure S4. Main results with polynomials of problematic demographics, 2021 (2SLS).

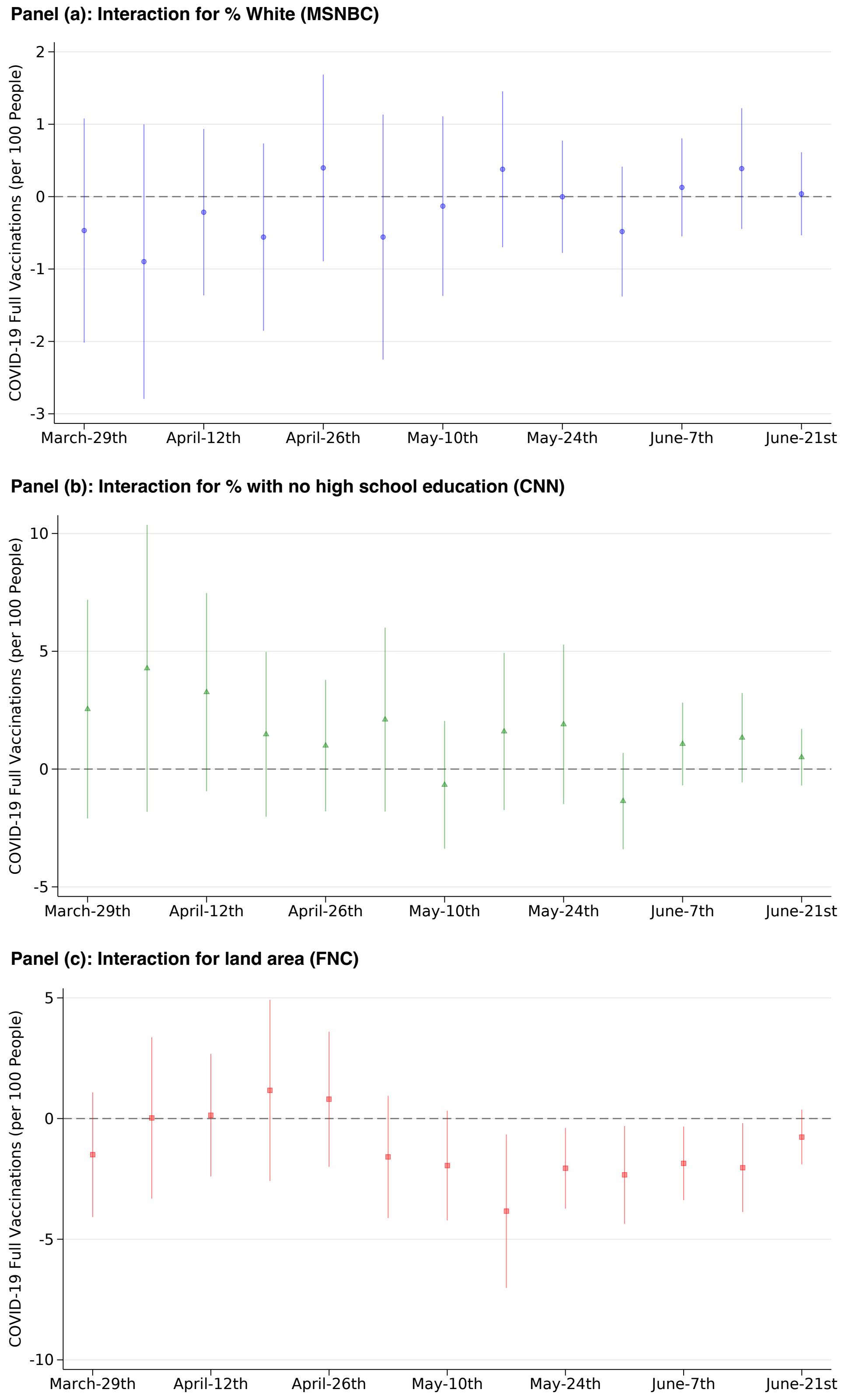

Figure S5. Main results adding interactions with viewership, for problematic demographics, 2021 (2SLS).

S3.3 Alternative IV approach

In the main results, the CNN and MSNBC viewership measures are instrumented with the relative channel position of the other two channels, respectively. Figure S6 presents the main results instrumenting directly with their channel position. While the first-stage F-statistic for the CNN model stays similar (F-statistic = 2.5), it significantly drops for the MSNBC model (F-statistic = 0.2).

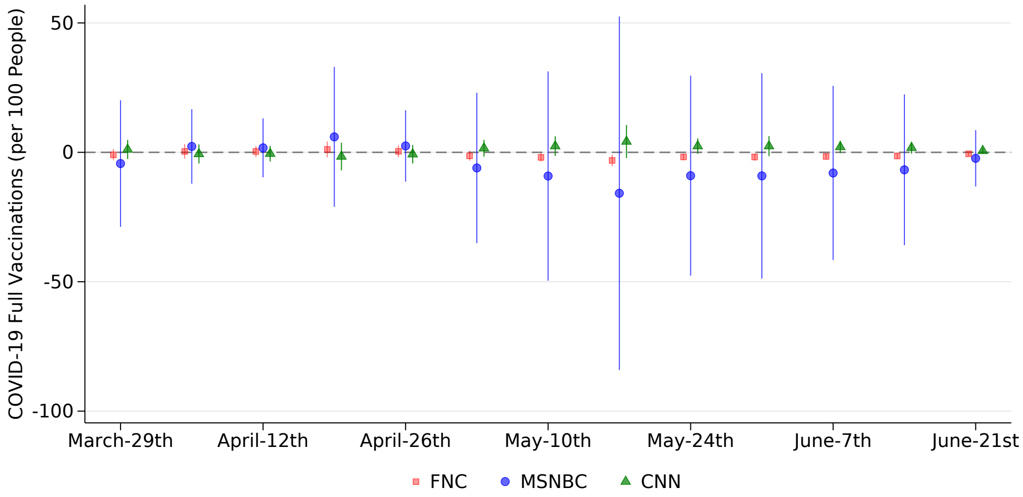

Figure S6. Main results using the same IV for all three networks, total population, 2021 (2SLS).

S4 Tables for main results

Table S5 presents the main results in a regression table format for all three channels separately.

	Network Viewership		
	FNC	MSNBC	CNN
March 29	-0.893	-0.206	1.136
	(1.067)	(0.639)	(1.086)
April 5	0.369	-0.780	1.863
	(1.379)	(0.939)	(1.583)
April 12	0.288	-0.187	1.597
	(1.050)	(0.741)	(1.077)
April 19	1.119	-0.630	0.467
	(1.526)	(0.854)	(1.159)
April 26	0.434	0.251	0.125
	(1.149)	(0.686)	(0.946)
May 3	-1.314	-0.202	1.797
	(0.946)	(0.733)	(1.255)
May 10	-1.934**	0.275	0.992
	(0.842)	(0.622)	(0.844)
May 17	-3.158***	0.823	1.039
	(1.100)	(0.675)	(0.700)
May 24	-1.803***	0.327	1.307
	(0.625)	(0.483)	(0.819)
May 31	-1.832**	-0.341	0.0364
	(0.694)	(0.479)	(0.569)
June 7	-1.636***	0.364	0.782
	(0.569)	(0.432)	(0.458)
June 14	-1.443**	(0.544)	0.601
	(0.596)	(0.507)	(0.428)
June 21	-0.508	0.0434	0.210
	(0.398)	(0.282)	(0.261)

Table S5. Effect of Cable News viewership on COVID-19 full vaccinations, 2021 (2SLS). Standard errors in parentheses. Significance levels: * $p < 0.10$, ** $p < 0.05$, *** $p < 0.01$.

S5 Other outcomes for all networks

Outcome	FNC	MSNBC	CNN
Vacc. Hesitancy	0.04^{+} (0.02)	-0.02 (0.01)	0.02 (0.02)
Outbreak Concern	0.01 (0.10)	0.07 (0.07)	-0.06 (0.10)
ICU Beds (#)	490 (1135)	-569 (384)	-138 (433)
Hospitals (#)	26 (41)	-19 (12)	-1 (15)
2016 Flu Vacc. (%)	-0.58 (7.46)	-0.11 (6.72)	9.34 (8.21)
2017 Flu Vacc. (%)	-0.45 (7.98)	0.05 (6.31)	9.82 (7.65)
2018 Flu Vacc. (%)	-0.05 (8.47)	-0.16 (6.36)	9.36 (8.58)

Table S6. Further outcome measures. Two Stages Least Squares (2SLS) estimates of the effect of each network's viewership on further outcome measures. Viewerships are instrumented using the lineup channel positions. Regressions include demographic and cable-system controls. Standard errors are clustered by state. Estimates are relative to a one s.d. increase in the network's viewership. $^{+}$ $p < 0.10$, * $p < 0.05$, and ** $p < 0.01$.

S6 Networks discourse

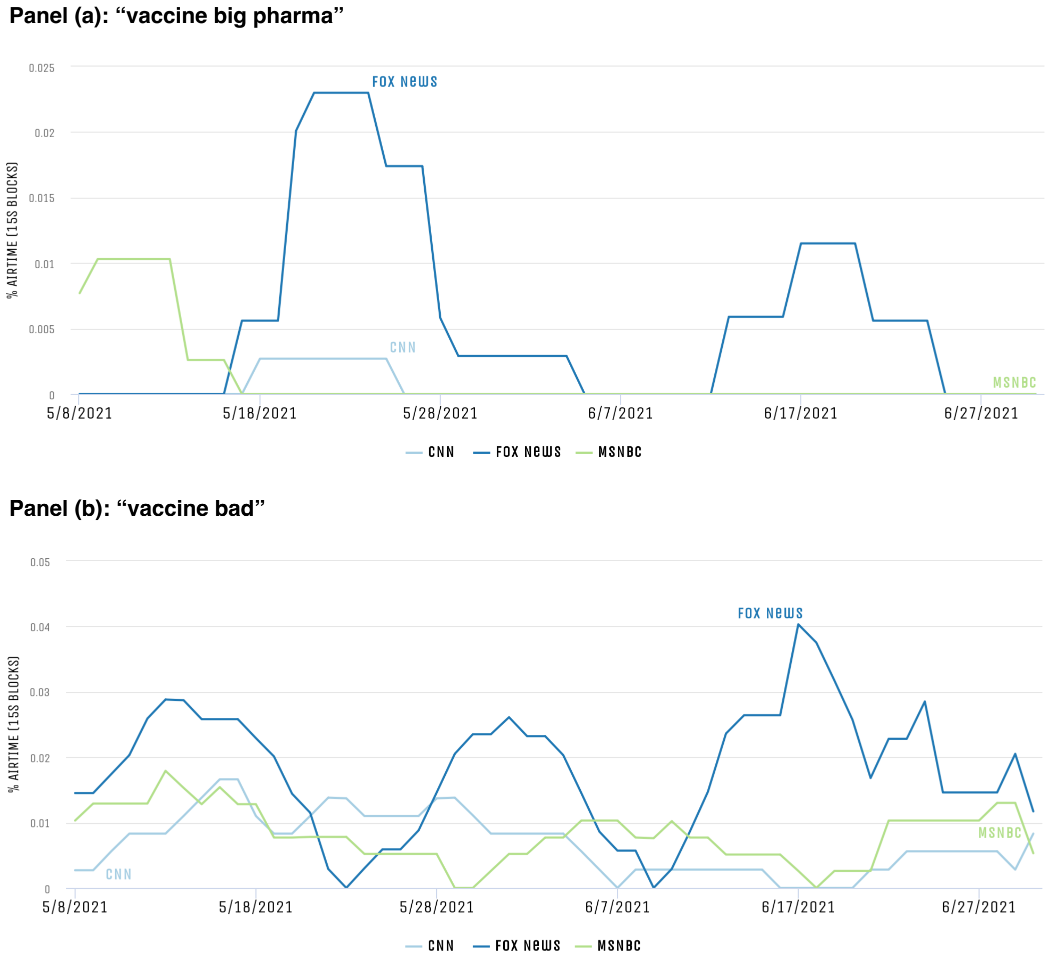

Figure S7. Divergent narratives on vaccines by network in May-June 2021. Each panel presents the smoothed frequencies of each phrase in the major news channels. The figure was produced using the GDELT Television Comparer API (accessible at `https://api.gdeltproject.org/api/v2/summary/summary`). The GDELT API is a flexible news search interface where one can specify specific networks and specific days to provide statistics on relative frequencies for user-provided search queries.

S7 Robustness checks

S7.1 OLS estimates for main results

Figure S8 reports the OLS estimates of the main results by age categories for all three channels.

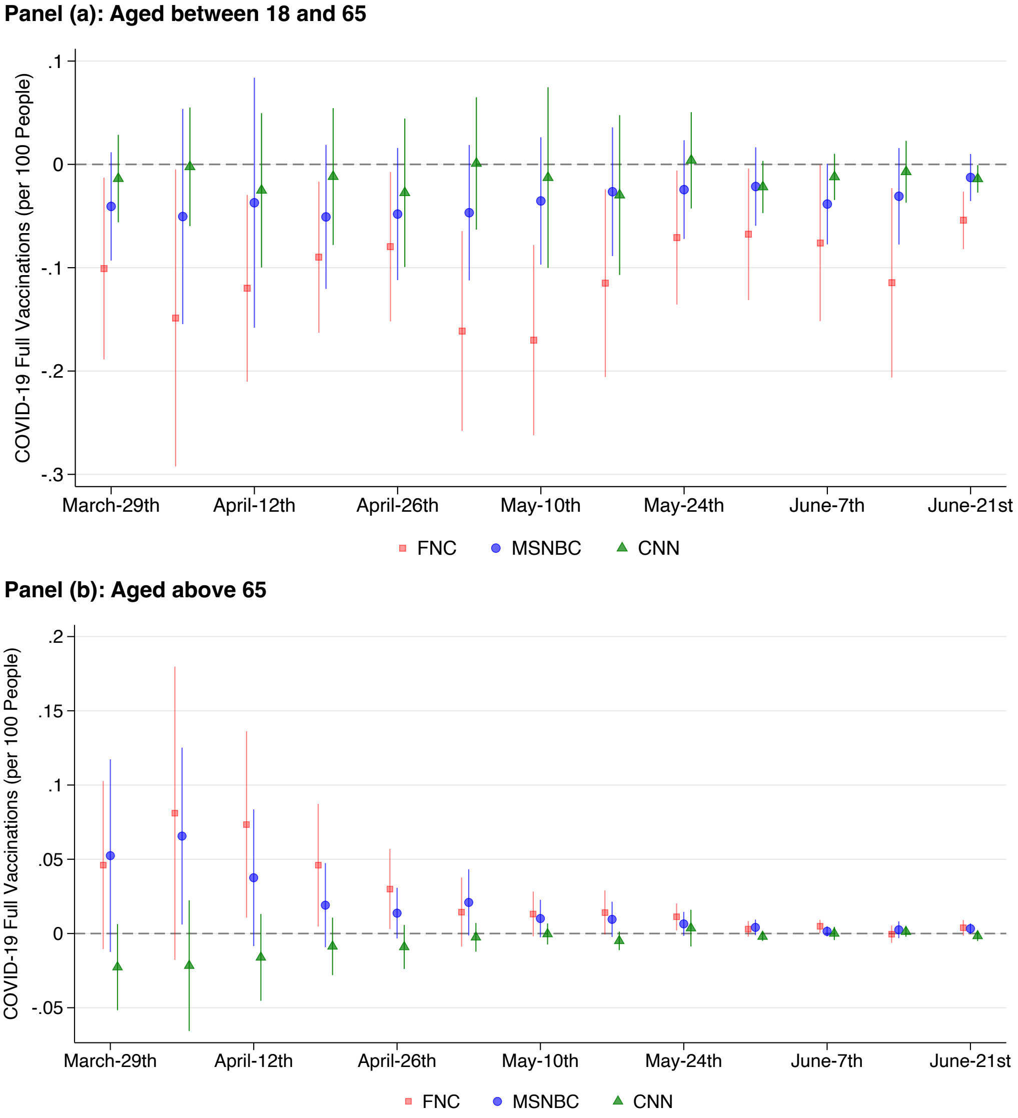

Figure S8. Effect of networks viewership by age category, 2021 (OLS).

S7.2 Results with extended period of analysis

In Figure S9, we show the results of the main model for all weeks of 2021 (starting with the week of January 11th, 2021). Since the geographical coverage of COVID-19 full vaccinations is smaller for early weeks, the county composition of regressions before our main results is not the same and affects instrument relevance.

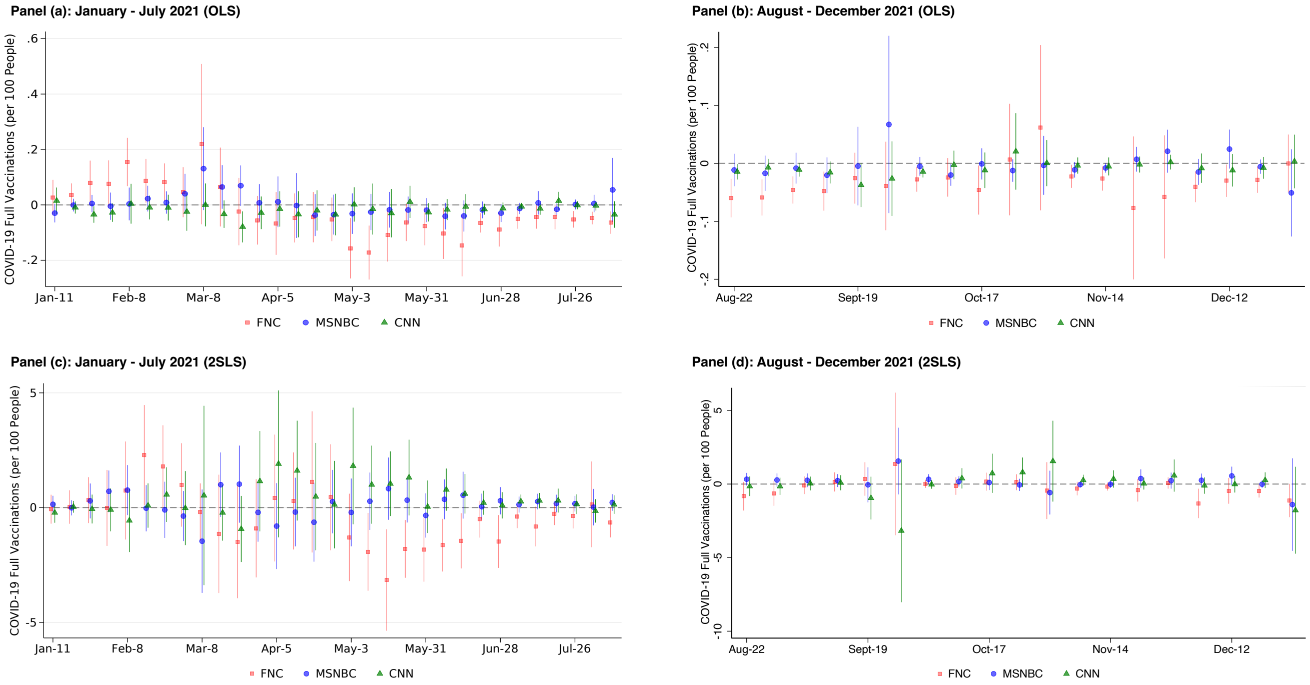

Figure S9. Main results including all weeks, total population, 2021.

S7.3 Partisanship and ideology checks

Figure S10 shows main results controlling for the Republican share at the 1992 and 1996 presidential elections. In Figure S11, we interact each presidential election variable with the respective channel position instrument.

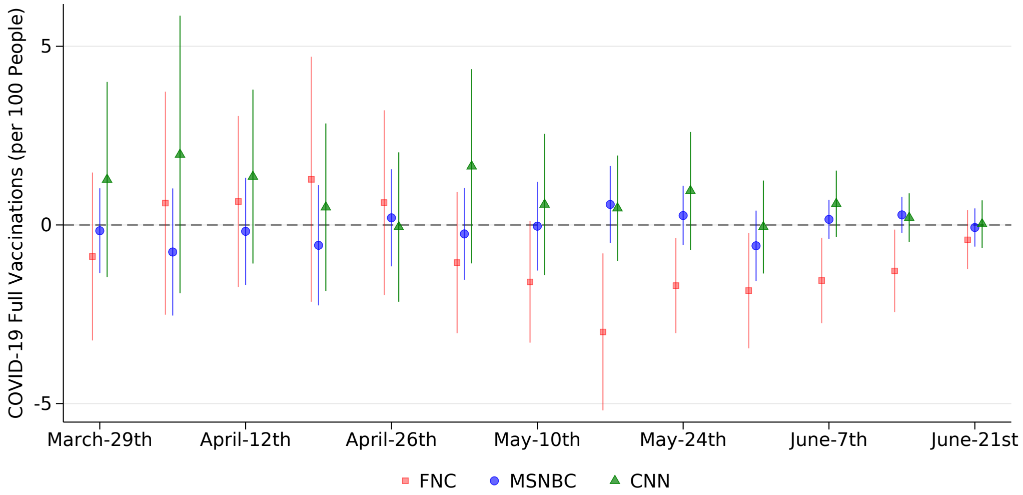

Figure S10. Main results with presidential elections controls, total population, 2021 (2SLS).

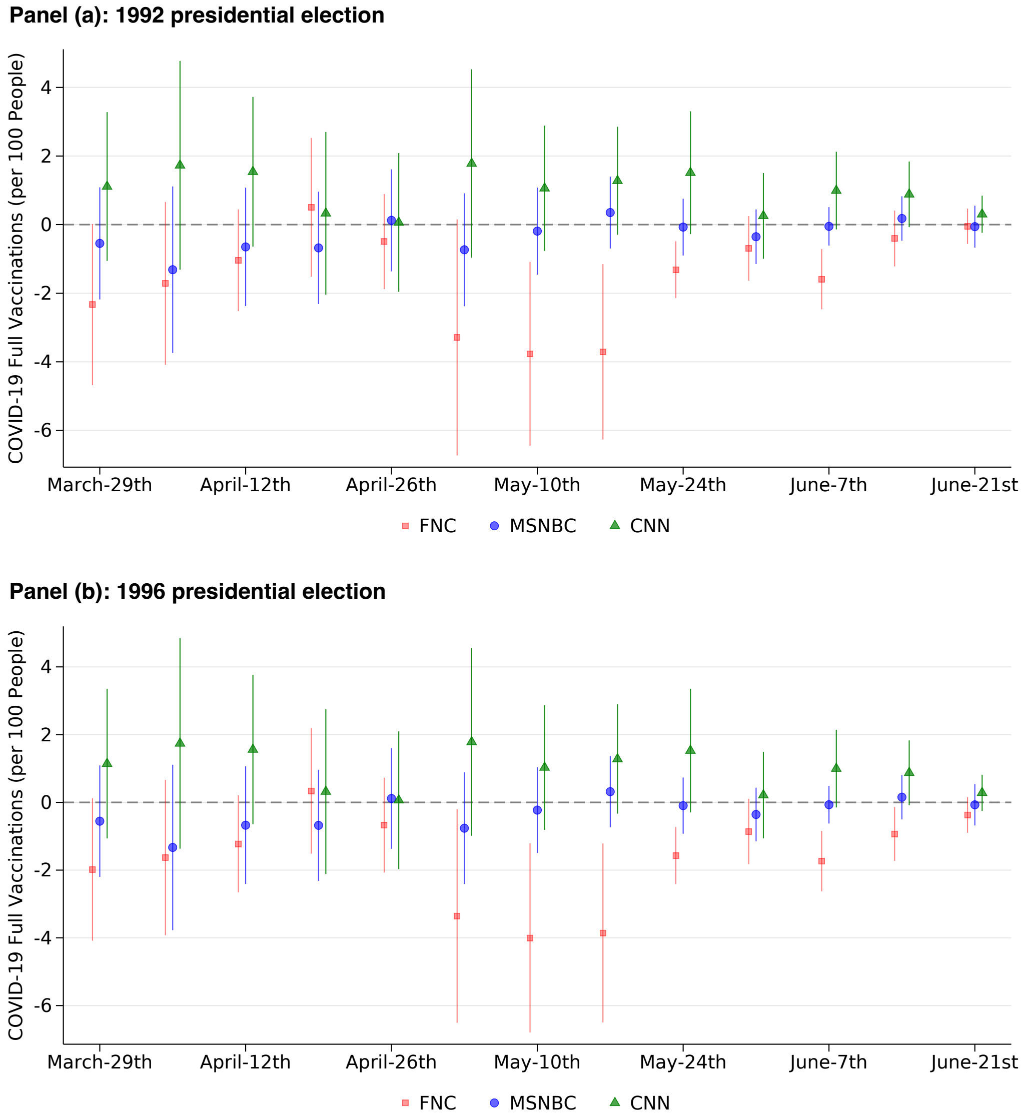

Figure S11. Main results interacting presidential election controls with media viewership, total population, 2021 (2SLS).

Figure S12 shows the main results controlling for the self-reported Republican and conservative affiliation from Gallup. We also interact those two estimates (2012-2019 and 2016-2019) with the respective channel position instrument in Figure S13.

Panel (a): 2016-2019 estimates

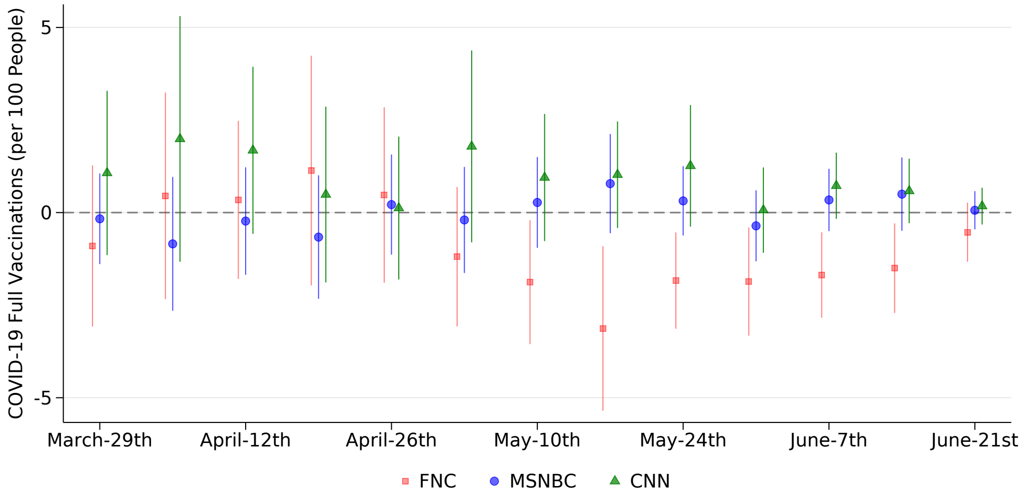

Panel (b): 2012-2019 estimates

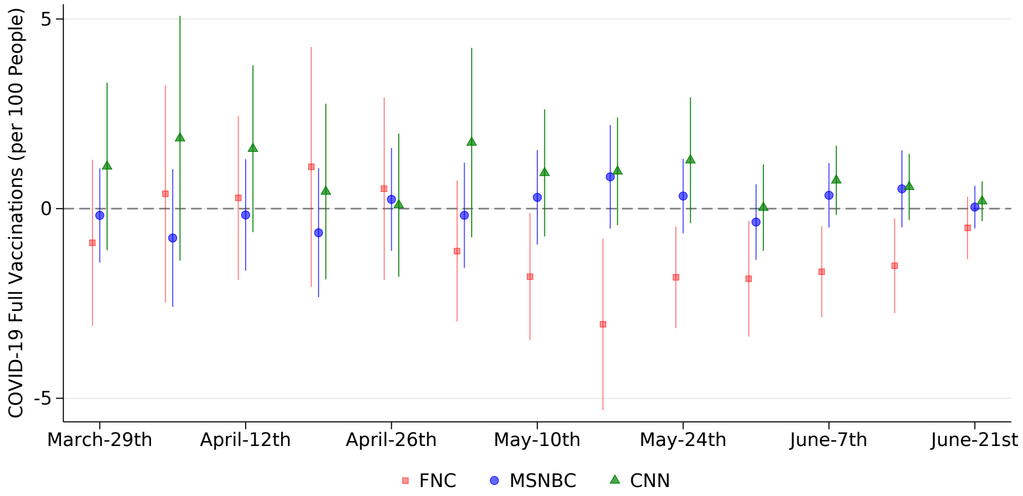

Figure S12. Main results controlling for self reported political affiliation (2016-2019), total population, 2021 (2SLS).

Panel (a): 2016-2019 estimates

Panel (b): 2012-2019 estimates

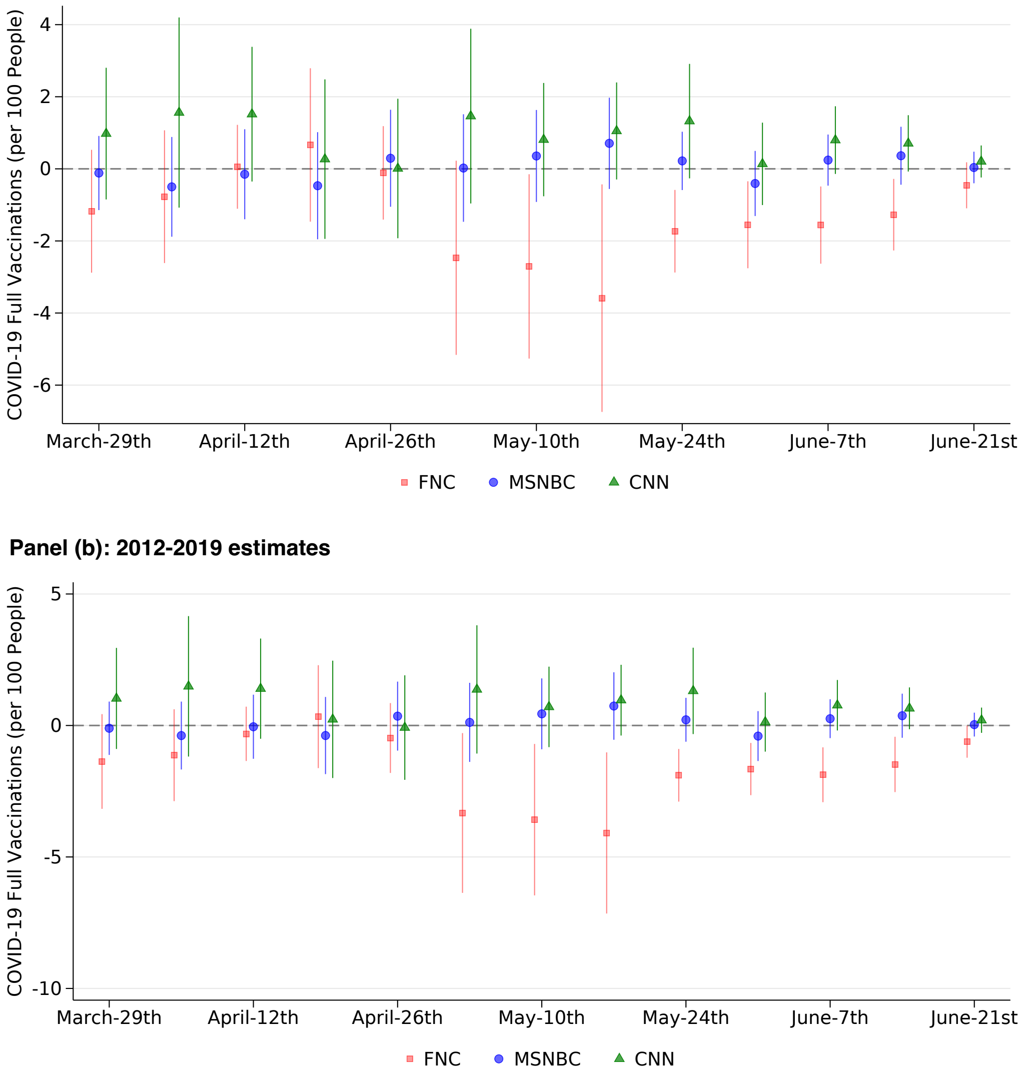

Figure S13. Main results interacting self reported political affiliation with channel position, total population, 2021 (2SLS).

S7.4 Television viewership checks

In Table S7, we use data on time spent watching TV (Average Time Use Survey) as an outcome in our analysis. Results show that the individual viewership of each network has no impact on the time spent watching TV. In Figure S14, we also show that our main results are robust to the inclusion of this variable as control considering the reduced sample size.

	# minutes spent on TV		
FNC Viewership	-4.080	—	—
	(41.73)		
MSNBC Viewership	—	2.643	—
		(12.94)	
CNN Viewership	—	—	-0.0250
			(22.76)
Observations	329	329	329
R^2	-0.006	0.002	0.000

Table S7. Daily television viewership as alternative outcome (2SLS). Standard errors in parentheses. * $p < 0.10$, ** $p < 0.05$, *** $p < 0.01$.

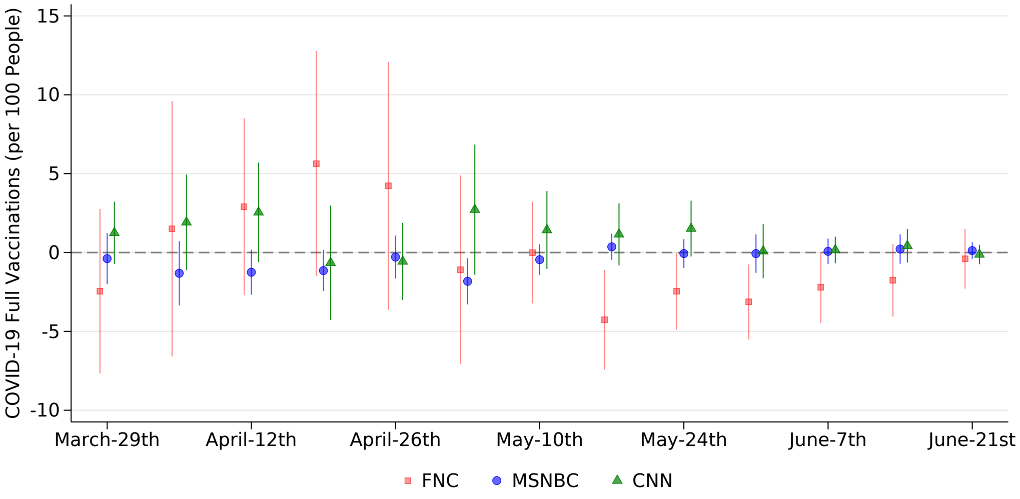

Figure S14. Main results including television daily viewership as control (2015-2019), total population, 2021 (2SLS).

S7.5 Health and health care sector checks

S7.5.1 COVID-19 cases and fatalities

Figure S15 shows our results controlling for COVID-19 cases and fatalities. In Panel (a), we control for cumulative confirmed cases and fatalities for the same week as the outcome. In Panel (b), we control for weekly new confirmed cases and fatalities lagged by four weeks compared to the outcome. We observe that results largely remain unchanged under these specifications. The same is true when interacting these measure with the instruments (cf. Figure S16).

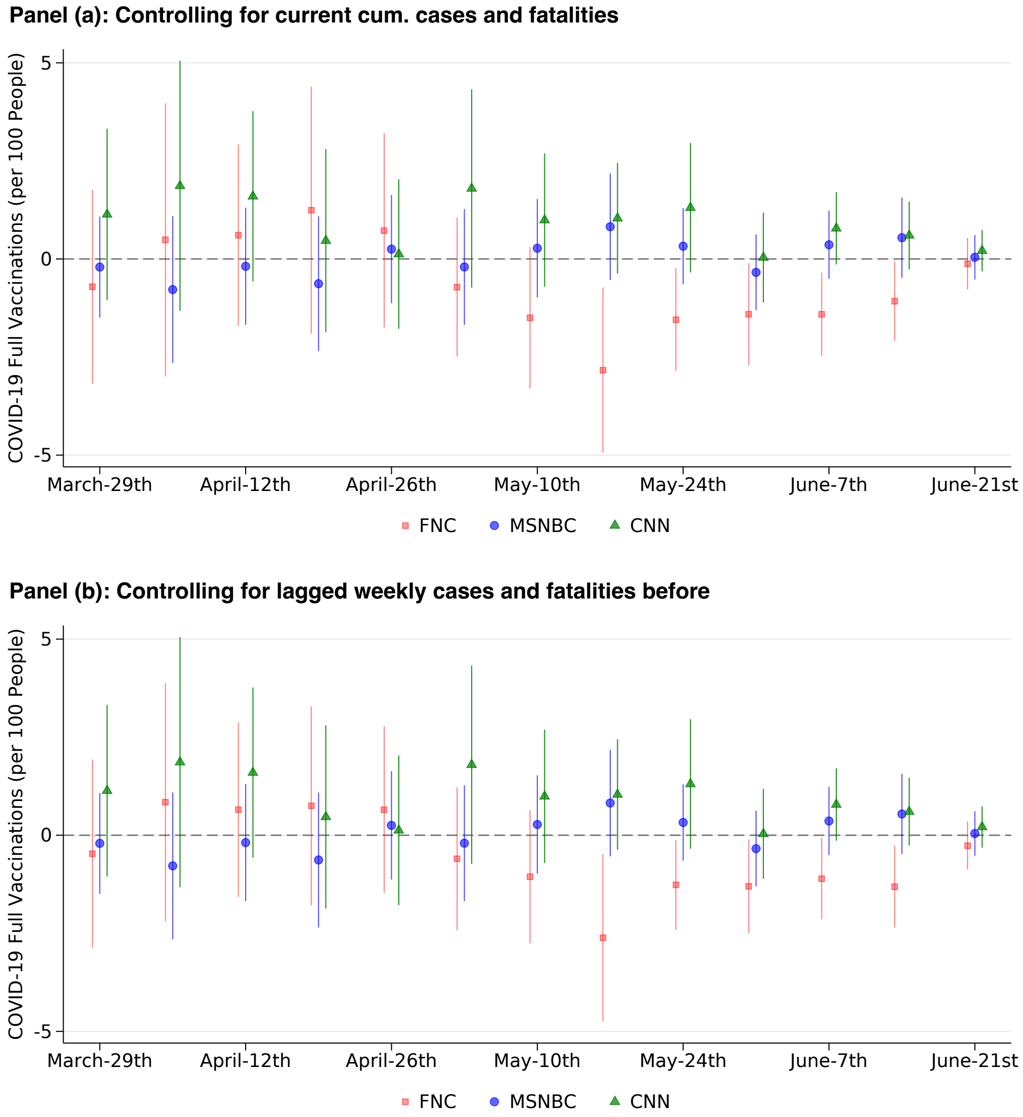

Figure S15. Main results controlling for cumulative COVID-19 cases and fatalities, total population, 2021 (2SLS).

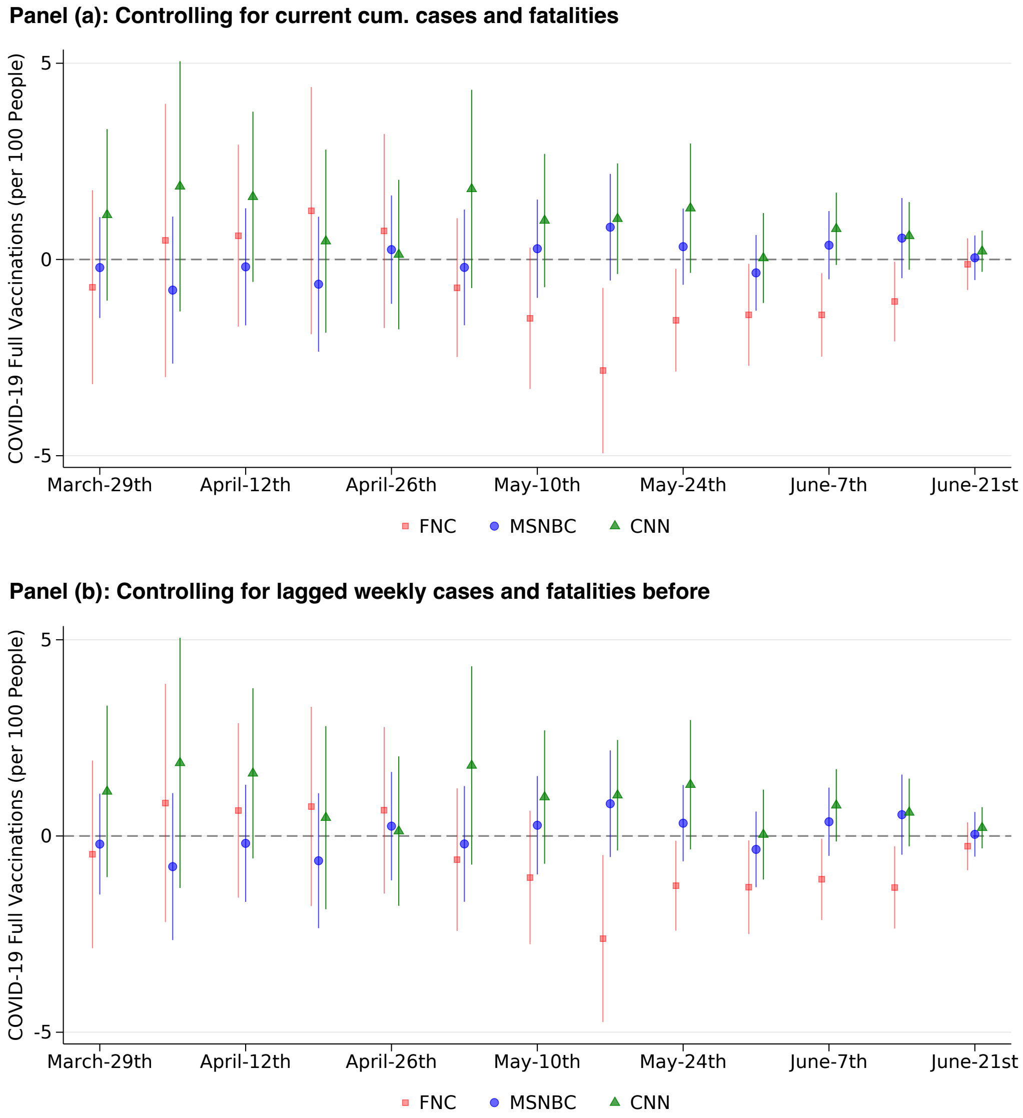

Figure S16. Interaction with instrument, total population, 2021 (2SLS).

S7.5.2 Vaccine hestiancy and outbreak risk

Table S8 reports the impact of viewership on vaccine hesitancy and the ability to handle a COVID-19 outbreak. When using those variables as outcomes in our main analysis, we observe a positive effect at the 10% level on vaccine hesitancy for FNC. When we instead included these variables as controls, we still see a negative and statistically significant effect on COVID-19 vaccinations for FNC after the week of May 10th (cf. Figure S17).

	Alternative COVID-19 Outcomes					
	% Hesitant	% Hesitant	% Hesitant	Outbreak concern	Outbreak concern	Outbreak concern
FNC Viewership	0.0369*	—	—	0.0131	—	—
	(0.0190)			(0.0960)		
MSNBC Viewership	—	-0.0167	—	—	0.0736	—
		(0.0121)			(0.0747)	
CNN Viewership	—	—	0.0215	—	—	-0.0598
			(0.0174)			(0.0995)
Observations	2996	2996	2996	2996	2996	2996
R^2	-1.634	-0.633	-0.899	-0.008	-0.244	-0.140

Table S8. Vaccine hesitancy and CVAC level of concern as alternative outcomes (2SLS). Standard errors in parentheses. Significance levels: * $p < 0.10$, ** $p < 0.05$, *** $p < 0.01$.

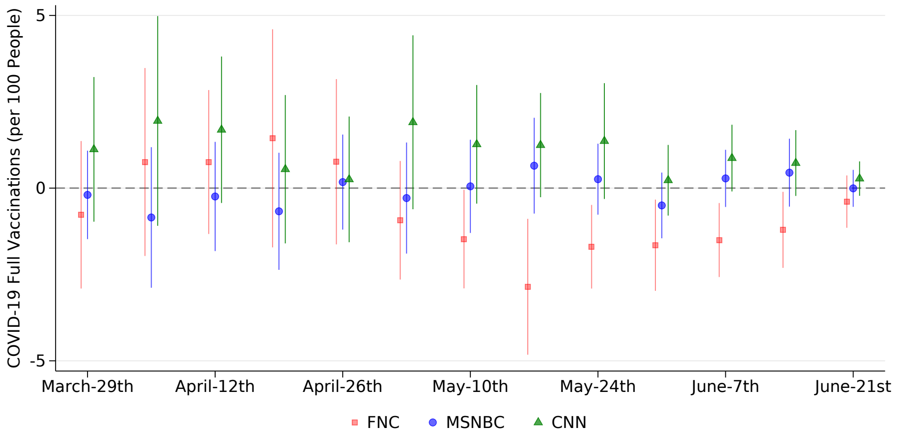

Figure S17. Main results controlling for COVID-19 vaccine hesitancy and CVAC level of concern, total population, 2021 (2SLS).

S7.5.3 Number of ICU beds and hospitals

Table S9 reports the impact of networks viewership on counties number of ICU beds and hospitals pre-COVID-19. Using the main model, we don't find any statistically significant coefficients for all three channels. Used as controls in the model from the main, on figure S18 we still see a negative and statistically significant impact on COVID-19 vaccinations for FNC after the week of May 10th.

	Health outcome					
	# ICU beds	# ICU beds	# ICU beds	# hospitals	# hospitals	# hospitals
FNC Viewership	490.0	—	—	25.97	—	—
	(1135.3)			(40.79)		
MSNBC Viewership	—	-569.2	—	—	-18.54	—
		(383.5)			(12.36)	
CNN Viewership	—	—	-137.5	—	—	-0.955
			(432.9)			(14.80)
Observations	2372	2372	2372	2372	2372	2372
R^2	-0.323	-0.786	-0.061	-0.820	-0.750	-0.005

Table S9. Number of ICU beds and hospitals as alternative outcomes (2SLS). Standard errors in parentheses. Significance levels: * $p < 0.10$, ** $p < 0.05$, *** $p < 0.01$.

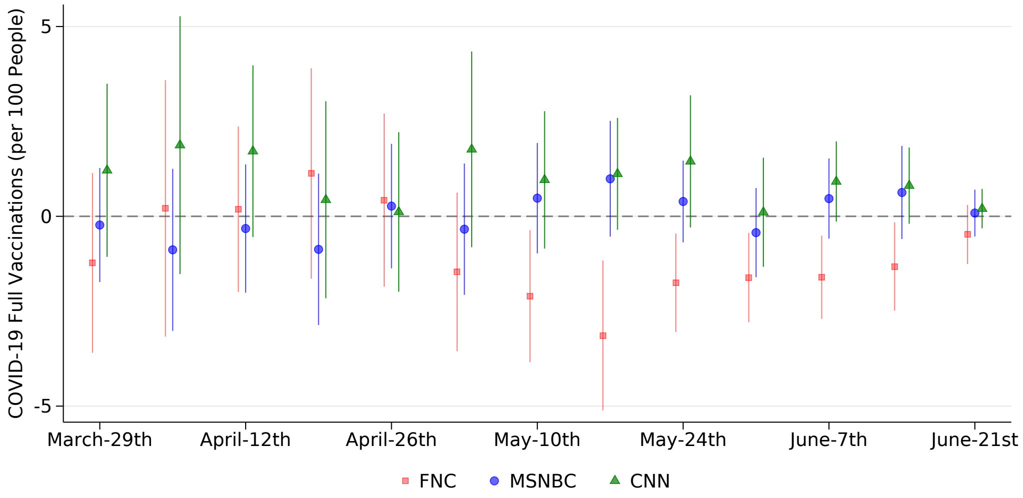

Figure S18. Main results controlling for ICU beds and hospitals, total population, 2021 (2SLS).

S7.5.4 Influenza vaccinations

Table S10 reports the effect of the networks viewership on counties share of influenza vaccination in 2016, 2017 and 2018. We do not observe a statistically significant effect for the three networks. Figure S19 show that our main results or robust to the inclusion of these variables as controls.

	Influenza vaccination year								
	2016	2016	2016	2017	2017	2017	2018	2018	2018
FNC Viewership	-0.579	—	—	-0.453	—	—	-0.0523	—	—
	(7.464)			(7.976)			(8.472)		
MSNBC Viewership	—	-0.114	—	—	0.0516	—	—	-0.159	—
		(6.718)			(6.307)			(6.365)	
CNN Viewership	—	—	9.344	—	—	9.825	—	—	9.359
			(8.211)			(7.653)			(8.585)
Observations	2994	2994	2994	2993	2993	2993	2993	2993	2993
R^2	-0.005	-0.001	-0.948	-0.004	0.000	-1.024	-0.000	-0.001	-0.919

Table S10. Share of influenza vaccination, 2016, 2017 and 2018 as alternative outcomes (2SLS). Standard errors in parentheses. Significance levels: * $p < 0.10$, ** $p < 0.05$, *** $p < 0.01$.

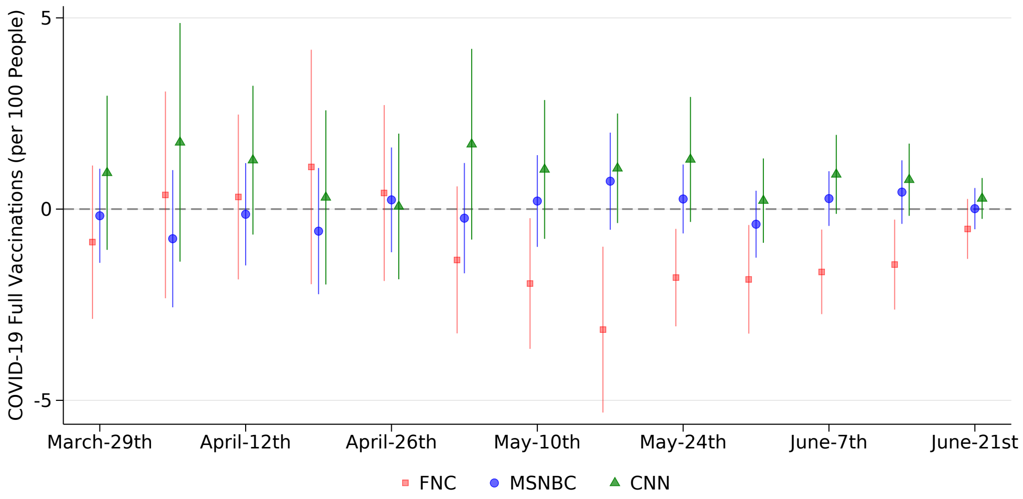

Figure S19. Main results controlling for 2016, 2017 and 2018 influenza vaccination, 2021 (2SLS).

S7.6 Specification checks

In Figure S20, we present the main results by excluding one control variable at a time (leave-one-out test). Therefore, 11 regressions are stacked on the same graph, leaving out each time one of the socio-demographics and political preferences controls from the main specification.

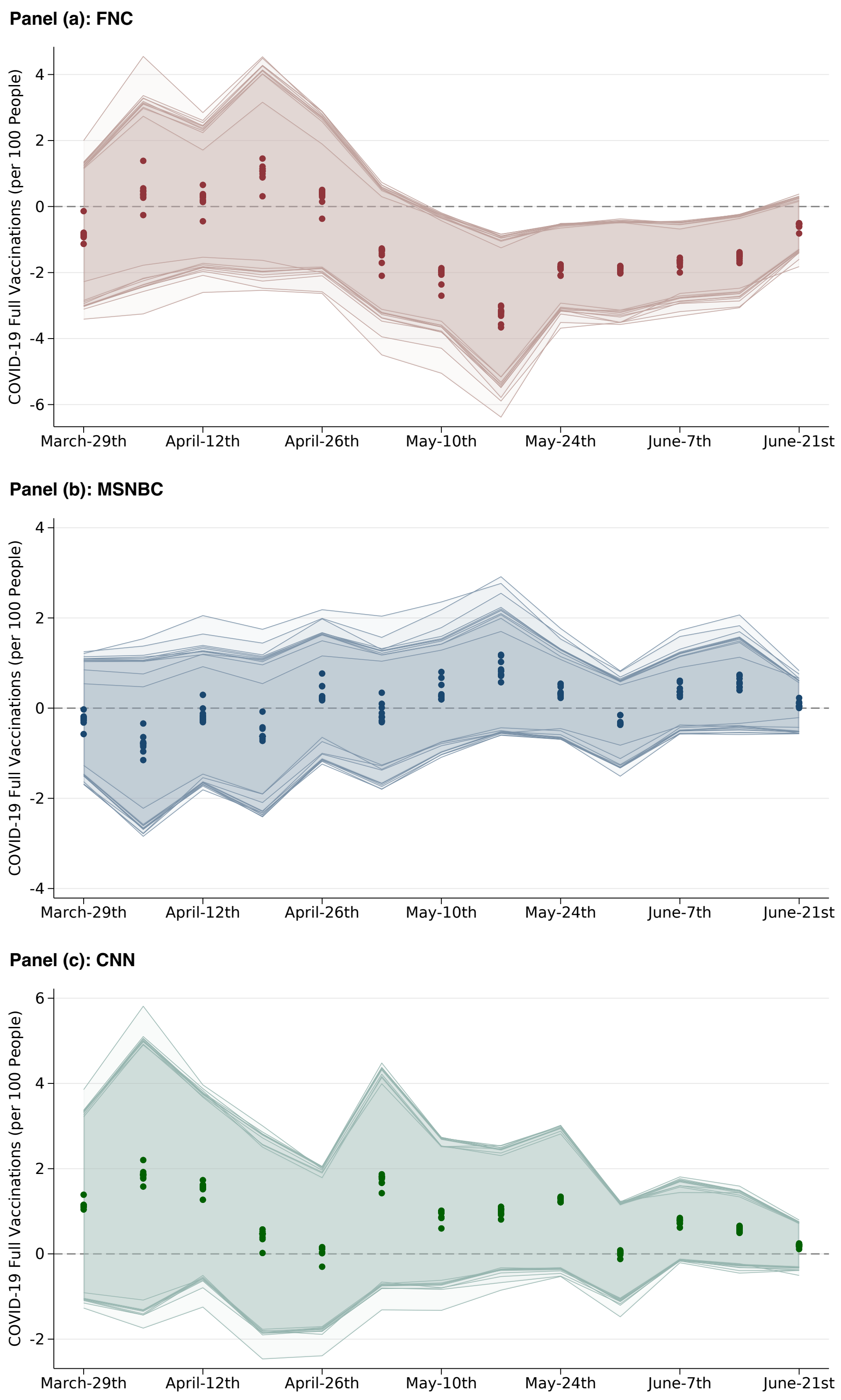

Figure S20. Leave-one-out test with control variables, total population, 2021 (2SLS).

Figure S21 shows the results using different sets of covariates. In Panel (a), we exclude the socio-demographic and political preferences controls, keeping only the controls for viewership and channel positions for the other networks. In Panel (b), we include a greater set of covariates: for ethnicities (a dummy for above-median hispanic population share and asian population share), education (proportion who attended high school), the share of population living in zip codes with access to the network, and a set of controls for the share of the population employed in different sectors: management and professional, services, sales and office, construction, production, arts/design/entertainment.

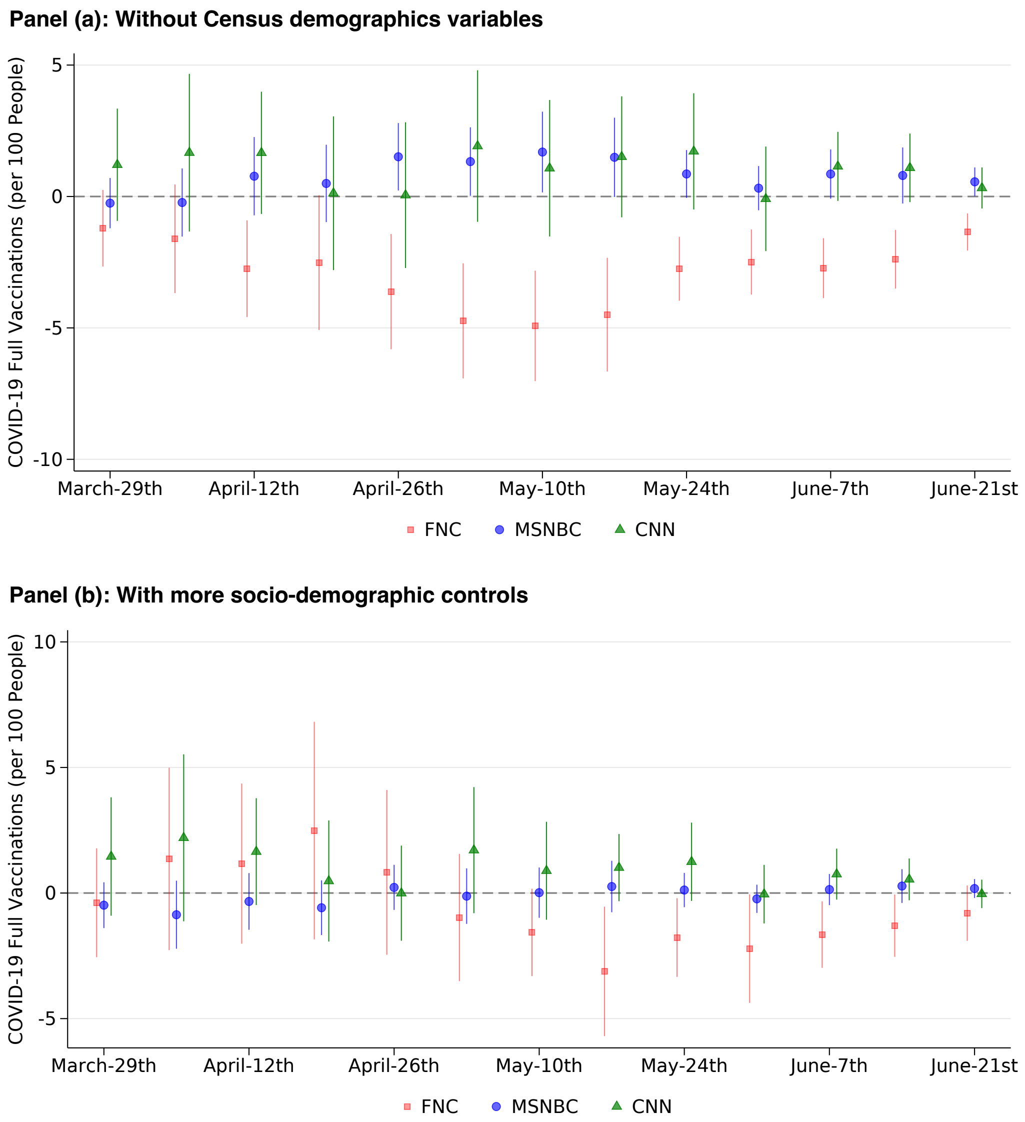

Figure S21. Main results controlling for different covariates, total population, 2021 (2SLS).

In Figure S22, we replace the Census Bureau occupation variables from Figure S21 Panel (b) with more recent occupational data from the American Community Survey (ACS), also provided by the Census Bureau. The survey includes share from the following sectors: medical, retail, agriculture, industrial and transport occupations.

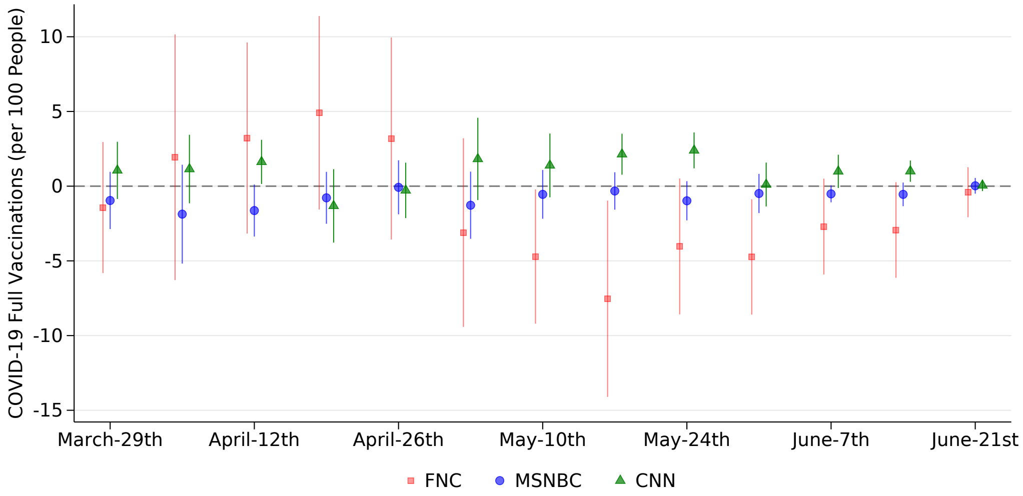

Figure S22. Main results with ACS occupation variables, total population (2SLS).

S7.7 Sample checks

In Figure S23, we present the main result following a perturbation test that excludes one of the 47 states at a time. Therefore, 47 regressions are stacked on the same graph, the dots represents estimates and the colored area represents the overlaid 95% confidence intervals.

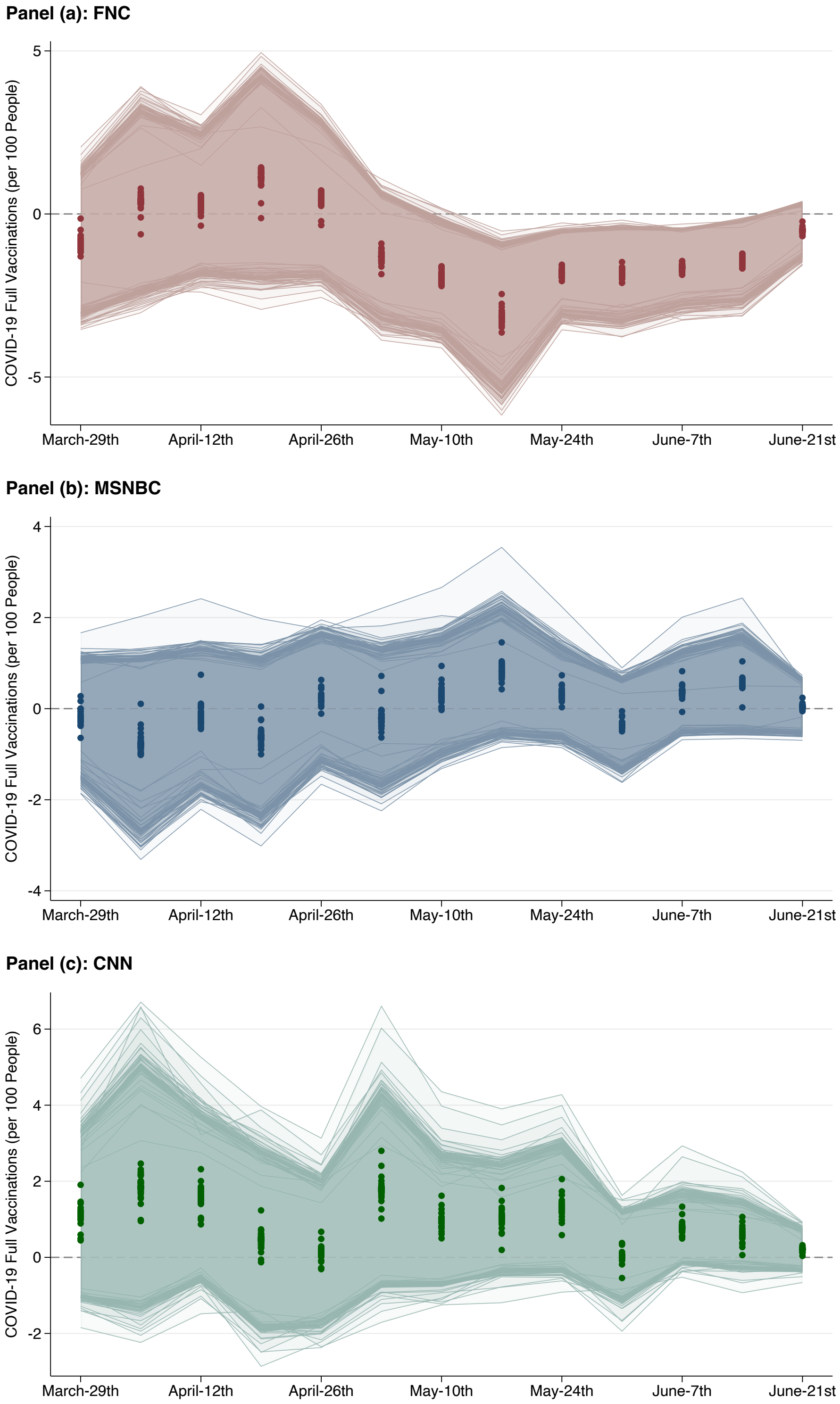

Figure S23. Perturbation test by leaving one state out, FNC channel, total population, 2021 (2SLS).

References

1. Schulte, F., Lucas, E., Rau, J., Szabo, L. & Hancock, J. Millions Of Older Americans Live In Counties With No ICU Beds As Pandemic Intensifies. *Kaiser Health News* (2020, accessed: 2021-11-02). https://khn.org/MTA2ODgzMw.
2. Bourassa, K. J., Sbarra, D. A., Caspi, A. & Moffitt, T. E. Social distancing as a health behavior: County-level movement in the United States during the COVID-19 pandemic is associated with conventional health behaviors. *Annals Behav. Medicine* **54**, 548–556 (2020).
3. Ash, E., Galletta, S., Hangartner, D., Margalit, Y. & Pinna, M. The Effect of Fox News on Health Behavior During COVID-19. *Available at SSRN 3636762* DOI: 10.31235/osf.io/abqe5 (2020).
4. Martin, G. J. & Yurukoglu, A. Bias in Cable News: Persuasion and Polarization. *Am. Econ. Rev.* **107**, 2565–99 (2017).
